# Systematic Review: The Relationship Between the Faecal Microbiome and Colorectal Neoplasia in Shotgun Metagenomic Studies

**DOI:** 10.1111/apt.70252

**Published:** 2025-08-12

**Authors:** Sarah Manning, Eleanor Hackney, Yashvee Dunneram, Mark A. Hull, Suparna Mitra, Christopher J. Stewart, Panayiotis Louca, Nick Meader, Linda Sharp, Colin Rees

**Affiliations:** ^1^ South Tyneside and Sunderland NHS Foundation Trust South Tyneside District Hospital South Shields UK; ^2^ Newcastle University, Population Health Sciences Institute, Newcastle University Centre for Cancer Newcastle Upon Tyne UK; ^3^ Human Nutrition and Exercise Research Centre, Centre for Healthier Lives, Population Health Sciences Institute Newcastle University Newcastle Upon Tyne UK; ^4^ Leeds Institute of Medical Research University of Leeds, St James University Hospital Leeds UK; ^5^ Newcastle University Clinical and Translational Institute Newcastle Upon Tyne UK

## Abstract

**Background:**

The human gut microbiome is of academic and clinical interest. Associations between certain organisms and colorectal neoplasia have been reported, but findings have limited reproducibility in different populations.

**Methods:**

We performed a systematic review of whole metagenome shotgun sequencing studies using faecal samples from patients with colorectal neoplasia and control populations. Searches were performed on 30th June 2023. We identified 26 studies, reporting on 22 study populations (13 from Asia, five from Europe and four from North America). Study size ranged from 14 to 971 individuals (mean 170).

**Results:**

Some reproducible data were identified, such as the significant enrichment of *Fusobacterium nucleatum* and *Parvimonas micra* in colorectal cancer patients compared to controls (in 10 and nine studies, respectively). However, 21 out of 26 studies scored poorly on quality appraisal, specifically surrounding selection of cases and controls. Definitions of controls varied; some studies used individuals with normal endoscopic investigations, some used ‘healthy’ individuals where no colonoscopy was performed, and one used those with non‐neoplastic findings (haemorrhoids). There was even less reproducibility of data in studies where individuals with colorectal polyps were compared to controls, possibly because of heterogeneity in these patient groupings as a variety of definitions for ‘polyp cases’ were used.

**Conclusions:**

Heterogeneity and potential for bias indicates that findings should be interpreted with caution. Standardised protocols to ensure robust methodology and allow pooling of large‐scale data are required before these findings can be used in clinical practice (PROSPERO: CRD42023431977).

## Graphical abstract

26 studies were identified, reporting on 22 populations. Size ranged from 14 to 971 individuals (mean 170). Some reproducible data were identified; *F. nucleatum* and *P. micra* were significantly enriched in colorectal neoplasia compared to controls in 10 and 9 studies respectively. However, evidence quality was poor, limiting the comparability of studies.

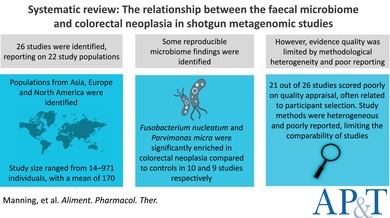

## 1 Introduction

The term ‘microbiome’ refers to the collective genome of the micro‐organisms living in a particular environment. The human microbiome consists of 10–100 trillion microorganisms, the vast majority of which are found in the gastrointestinal tract [1]. These organisms are not simply bystanders, they have evolved a symbiotic relationship with their human hosts over thousands of years and are involved in multiple complex organism‐host interactions [2]. The human gut microbiome is implicated in many disease processes [3] and the development of a number of cancers [4], including colorectal cancer (CRC) [5].

Globally, there were nearly 2 million new cases of CRC in 2022, and > 900,000 deaths [6]. The majority of these cancers develop over several years from pre‐cancerous lesions, such as adenomas or serrated lesions. Identifying these lesions at an early stage provides an opportunity to prevent patients from developing cancers, reducing the morbidity and mortality of CRC [7].

Studies have suggested that the composition and diversity of the faecal microbiome may be altered in patients with colorectal neoplasia when compared to controls [8, 9], with increased abundance of certain taxa (such as *Fusobacterium nucleatum* ) having been described [10]. Differences have also been shown between early and late neoplastic stages [11]. However, mechanisms and causation are still unclear [12], and there remains a lack of consensus on specific microbial signals, with a variety of associations reported in the literature. Studies generally have small sample sizes and employ heterogeneous methods [9]. As a result, while promising predictive models have been developed, their reproducibility and clinical utility may be constrained [13].

The gold standard test for colorectal neoplasia detection is colonoscopy; however, non‐invasive tests, such as the faecal immunochemical test (FIT), are now being widely used in both screening and symptomatic populations to stratify the need for endoscopic procedures. Colonoscopy is potentially uncomfortable and is associated with considerable anxiety [14], as well as other risks (such as bowel perforation, bleeding, adverse drug reactions, and death [15]). Many healthcare services are struggling to deliver adequate colonoscopy capacity. Improved risk stratification with non‐invasive tests, including potential microbiome‐based neoplasia biomarkers, is of significant interest.

As well as FIT, other stool protein‐based biomarkers have been shown to predict colorectal neoplasia [16], but the majority are markers of blood in stool, meaning that they have limited additional benefit [9]. Microbial markers could be used in conjunction with FIT, increasing diagnostic performance, with some studies finding that predictive models combining FIT and stool microbial markers had superior sensitivity for CRC and advanced adenoma to FIT alone [17, 18].

The primary aim of this systematic review was to summarise evidence on faecal shotgun metagenomic features (alpha diversity, beta diversity and taxonomic associations) associated with colorectal neoplasia. In addition, we sought to explore whether any distinct faecal microbiome changes have been reported in association with different types of colorectal neoplasia for example, polyps compared to cancer or adenomas compared to serrated lesions.

A previous systematic review by Yu et al. [9] reviewed articles published up until December 2020, including all studies that explored associations between microbiota and colorectal neoplasia or developed and validated predictive models. Consequently, it encompassed studies employing different technologies for microbiome analysis and diverse sample types. There has been a rapid evolution of the evidence base in this field, and growing use of whole metagenome shotgun sequencing (subsequently referred to as ‘shotgun sequencing’ in this review). Shotgun sequencing includes all DNA present in a sample, compared to older amplicon sequencing which involves the amplification of a specific marker gene, such as the 16 s rRNA gene in bacteria and archaea. This means that shotgun sequencing offers multiple advantages over 16 s rRNA amplicon sequencing (e.g., detection of organisms to species and strain level, detection of non‐bacterial organisms and the identification of potential gene functions). Therefore, it is timely to undertake a contemporary review of studies, specifically those using stool samples and performing shotgun sequencing.

## 2 Methods

The review was registered with the International Prospective Register of Systematic Reviews (PROSPERO) [19] (CRD42023431977) and has been conducted and reported in line with the Preferred Reporting Items for Systematic Review and Meta‐Analysis Protocols (PRISMA) 2020 statement [20]. For full detailed methods, including all exclusions, see Appendix S1.

To be eligible for inclusion, papers needed to report an original shotgun sequencing study comparing human faecal microbiome findings from individuals with colorectal neoplasia to those from control populations.

We suspected that more widespread use of newer technologies since the last review by Yu et al. [9] (e.g., shotgun sequencing compared to 16 s rRNA amplicon sequencing) may result in new or different findings. Whilst we included amplicon sequencing studies in our PROSPERO [19] registration, as our study progressed it became apparent that there was sufficient data in the shotgun studies alone. Therefore, we decided to publish these as two separate review articles so that findings could be compared more easily. Studies which reported findings from both shotgun and amplicon sequencing were included here if the data were presented separately. Only data from shotgun sequencing were included in the synthesis for this article. A separate review article of amplicon sequencing studies is planned by the authors.

Studies comparing faecal and tissue samples suggest that these are significantly different and therefore cannot directly be compared [21]. Also, due to the invasive nature of sampling, tissue samples cannot easily be used for population screening. For these reasons, we chose to only include studies using stool samples.

Articles studying patients with neoplasia associated with either inflammatory bowel disease (IBD) or genetic predispositions, such as Lynch syndrome, were excluded as these conditions are likely to have different aetiologies and are therefore beyond the scope of this review.

Searches were conducted from database inception to 30th June 2023 in MEDLINE (via OVID), EMBASE (via OVID), Web of Science, and SCOPUS. The PICO and associated search strategies can be seen in Appendix S1.

Abstract screening was done using Rayyan [22] in a double‐blind manner. All abstracts were screened by at least two of four reviewers (Sarah Manning (SMa), Suparna Mitra (SMi), Eleanor Hackney (EH) and Yashvee Dunneram (YD)). The lead reviewer (SMa) screened all abstracts, with SMi, EH and YD screening approximately one third each. If an abstract was considered potentially eligible by at least one reviewer, the full text was retrieved. Full text screening was done in the same fashion. Articles which were considered by both reviewers to meet the inclusion criteria were included in the final synthesis. Any disagreements were discussed by the group of reviewers and unanimous decisions were reached for all articles.

A data extraction form was created (Table S3). Three reviewers (SMa, EH, YD) extracted data from one third of the studies each. These extractions were then checked for accuracy by another reviewer. To ensure that the lead reviewer had oversight of all data, SMa checked extractions done by EH and YD.

Quality appraisal was undertaken using the Newcastle‐Ottawa tool [23] and was performed and checked by SMa, EH, and YD in the same fashion.

A narrative synthesis was presented according to SWiM guidelines by Campbell et al. [24]. This was presented in the following format:

1. Bacterial findings:
  - Diversity in ‘cases’ (individuals with CRC and/or colorectal polyps) compared to controls:
    - Alpha diversity—observed richness, Shannon and/or Simpson indices were accepted as all studies used one or more of these measures. These were displayed in a harvest plot so that study findings could be more easily compared, while bearing in mind quality appraisal scores.
    - Beta diversity—any reported measure was accepted due to wide variation within studies.
  - Bacterial associations—significantly enriched or depleted organisms (adjusted *p* value/false discovery rate (FDR) < 0.05) at species level:
    - In individuals with CRC compared to controls (species identified by ≥ 3 studies were reported).
    - In individuals with polyps compared to controls (species identified by ≥ 2 studies were reported).
    - In individuals with polyps compared to individuals with CRC (species identified by ≥ 2 studies were reported).
2. Non‐bacterial findings—diversity (any metric) and taxonomy (significantly enriched species) of viruses, fungi and archaea.

Given that this is a novel area and there is no established convention for reporting this type of data, a pragmatic decision was made by the team to report species identified by either ≥ 3 or ≥ 2 studies (for CRC and polyps respectively). This decision was based on the total number of species identified once data extraction was completed. The threshold was lower for polyps given the lower volume of evidence.

If findings were not reported to species level these were not included in data synthesis. Data on gene functions were reported by some studies but were not included in the final synthesis; it became clear during the process that the extraction and synthesis of these data was complex and unfeasible. We acknowledge that this is a deviation from the published protocol.

A small number of studies made attempts to validate their findings in other cohorts or test the predictive performance of specific markers, however, since this was done inconsistently, only the data from the original discovery cohorts were included in data synthesis.

The lack of conventional effect estimates in these types of data meant that quantitative meta‐analysis was not feasible; therefore, no statistical combination of study findings was performed.

## 3 Results

### 3.1 Study Selection

**FIGURE 1:**
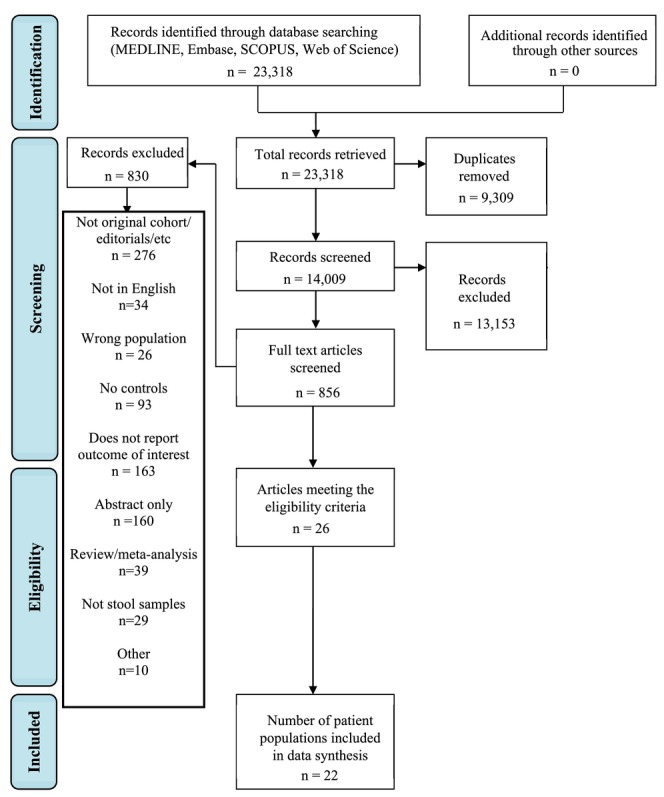
PRISMA [20] flow diagram.

Database searches found 23,318 results. Once duplicates were removed, 14,009 titles and abstracts were screened, with 856 full text articles assessed for eligibility. Finally, 26 papers reporting 22 patient populations were included. This is shown in the PRISMA [20] diagram (Figure 1).

### 3.2 Study Characteristics

Table 1 describes the 26 articles and 22 patient populations. There were 13 populations from Asia (nine from mainland China, two from Hong Kong, one from India and one from Japan), five populations from Europe (two from France, one from Austria, one from Italy and one from Norway) and four populations from North America (one containing individuals from both Canada and the USA, with three from the USA only). The largest two studies (one from the USA [50] and one from Japan [11]) included totals of 971 and 578 individuals respectively, including those with neoplasia and the comparators. The rest were much smaller (ranging from 14 to 386) and the mean number of participants from all studies was 170.

**TABLE 1:** List of included studies.

Patient population and location		Year	Authors	Organism types described	No. of controls	Control definition	No. of polyp cases	Polyp case definition	No. of CRC cases	Inclusion criteria/source of participants
Asia	**Population 1:** Hong Kong (Special Administrative Region of China)	2017	Yu, J et al. [25]	Bacteria	54	Patients with no neoplasia at colonoscopy	0	N/A	74	Mixed symptomatic and asymptomatic population—individuals attending for colonoscopy for assessment of lower GI symptoms or neoplasia screening
		2018	Nakatsu, G et al. [26]	Viruses	92 (38 additional controls recruited)					
		2019	Coker, OO et al. [27]	Fungi					73 (one case excluded – reason not reported)	
		2020	Coker, OO et al. [28]	Archaea						
	**Population 2:** Bhopal & Kerala (India)	2019	Gupta, A et al. [29]	Bacteria	30	‘Healthy’ patients – no colonoscopy performed	0	N/A	30	Cases: newly diagnosed CRC patients (indications for index colonoscopy not reported) Controls: healthy participants from a previous study [30]
	**Population 3:** Tokyo (Japan)	2019	Yachida, S et al. [11]	Bacteria	251	Patients with no CRC and ≤ 2 low risk adenomas (≤ 5 mm with no HGD) at colonoscopy	67	Patients with ≥ 3 adenomas or ≥ 1 advanced adenoma (≥ 5 mm or with HGD), Patients with serrated lesions were excluded	258—Stage 0: 73 Stage I: 75 Stage II: 3 Stage III: 52 Stage IV: 22	Patients undergoing colonoscopy (indication for colonoscopy not reported)
	**Population 4:** Shanghai (China)	2020	Gao, R et al. [31]	Bacteria	45	Patients with no neoplasia at colonoscopy	23	Patients with ≥ 1 adenoma	32	Patients who had undergone colonoscopy and were subsequently classified as CRC, polyps or controls (no other details reported)
	**Population 5:** Chongqing (China)	2020	Yang, J et al. [32]	Bacteria	55	Cohabiting family members – no colonoscopy performed	0	N/A	52	Cases: CRC patients (indication for index colonoscopy not reported) Controls: Family members of cases who have lived with the patient for at least one year
	**Population 6:** Hainan (China)	2021	Chang, HB et al. [33]	Bacteria	12	Patients diagnosed as ‘healthy’ after colonoscopy	0	N/A	6	Asymptomatic population–individuals attending for screening colonoscopy
	**Population 7:** Shanghai (China)	2021	Gao, R et al. [34]	Bacteria Viruses	71	Patients with no neoplasia at colonoscopy	63	Patients with ≥ 1 adenoma	91	Mixed asymptomatic and symptomatic population–individuals undergoing colonoscopy for symptoms of suspected of colorectal cancer or asymptomatic individuals undergoing screening colonoscopy
		2022	Gao, R et al. [35]	Fungi						
	**Population 8:** Zhengzhou (China)	2021	Liu, YF et al. [36]	Bacteria	6	Patients with haemorrhoids at colonoscopy	6	Patients with ≥ 1 adenoma	4	‘Hospitalised patients’ (no other details reported)
	**Population 9:** Shanghai (China)	2021	Yang, YZ et al. [37]	Bacteria	100 (50 young, 50 old)	Not reported	0	N/A	100 (50 young onset, 50 old onset)	Not reported
	**Population 10:** Hong Kong (Special Administrative Region of China)	2022	Coker, OO et al. [38]	Bacteria	128	Patients with no neoplasia at colonoscopy	140	Patients with ≥ 1 adenoma	118	Individuals undergoing ‘standard’ colonoscopy (no further details reported)
	**Population 11:** Wuhan (China)	2022	Zhang, J et al. [39]	Bacteria Viruses	35	Patients diagnosed as ‘healthy’ after physical examination	29	Patients with ≥ 1 adenoma	30	Patients with histologically confirmed colorectal adenoma/CRC or ‘healthy’ individuals attending the hospital (no other details reported)
	**Population 12:** Guangzhou (China)	2023	Lv, MW et al. [40]	Bacteria	71	Not reported	31	Patients with ≥ 1 TA	0	Individuals aged 40–80 years who were ‘high risk’ for CRC (no other details reported)
	**Population 13:** Shanghai (China)	2023	Zhang, H et al. [41]	Bacteria	6	Not reported	0	N/A	8	Not reported
Europe Europe	**Population 14:** Créteil (France)	2014	Zeller, G et al. [42]	Bacteria	61	Patients with no neoplasia at colonoscopy	42	Patients with ≥ 1 adenoma	53	Mixed asymptomatic and symptomatic population – individuals undergoing screening colonoscopy for average or higher‐than‐average CRC risk or for the assessment of lower GI symptoms/anaemia
	**Population 15:** Salzberg (Austria)	2015	Feng, Q et al. [43]	Bacteria	63	Patients with no neoplasia at colonoscopy	47	Patients with ≥ 1 advanced adenoma (villous or tubulovillous features, size ≥ 1 cm, or HGD)	46	Mixed asymptomatic and symptomatic population–individuals attending for colonoscopy as part of national CRC screening programme or for symptoms of suspected CRC
	**Population 16:** Vercelli (Italy)	2019	Tarallo, S et al. [44]	Bacteria	24	Patients with no neoplasia or IBD at colonoscopy	27	Patients with ≥ 1 adenoma	29	Individuals undergoing colonoscopy (indication not reported)
	**Population 17:** Nantes (France)	2020	Touchefeu, Y et al. [45]	Bacteria	20	Not reported	0	N/A	21	Cases: Individuals with histologically proven CRC, aged between 45 and 80 years (indication for colonoscopy not reported) Controls: Not reported
	**Population 18:** Oslo & Telemark (Norway)	2023	Bucher‐Johannessen, C et al. [46]	Bacteria	22	Patients with no findings at sigmoidoscopy	18	Patients with ≥ 1 high risk adenoma (≥ 10 mm, HGD or villous components) or ≥ 3 total adenomas	7	Asymptomatic population–participants in the NORCCAP study (healthy 50–64 year olds invited for screening sigmoidoscopy as part of the study)
North America	**Population 19:** Irvine (USA)	2022	Avelar‐Barragan, J et al. [47]	Bacteria	9	Patients with no neoplasia at colonoscopy	29	Patients with ≥ 1 polyp of any type (mixture of TA, hyperplastic, SSL and ‘other/unknown’ – handled as one group)	0	Asymptomatic population–individuals undergoing colonoscopy for neoplasia screening/surveillance
	**Population 20:** Toronto (Canada) and Boston, Houston & Ann Arbor (USA)	2018	Hannigan, GD et al. [48]	Bacteria Viruses	30	Patients with no abnormalities at colonoscopy	30	Patients with ≥ 1 adenoma	30	Cases: Individuals who had attended for colonoscopy and had histologically confirmed CRC/polyps (indication for colonoscopy not reported) Controls: Healthy individuals recruited from the community (colonoscopy purely for research purposes)
	**Population 21:** Washington DC (USA)	2016	Vogtmann, E et al. [49]	Bacteria	52	Patients awaiting surgery for non‐GI and non‐malignant conditions (no colonoscopy performed)	0	N/A	52	Cases: Newly diagnosed adenocarcinoma of the colon or rectum (indication for colonoscopy/other investigation not reported) Controls: Age and sex matched controls who were waiting surgery for non‐oncological and non‐gastrointestinal conditions (no colonoscopy performed)
	**Population 22:** Boston, USA	2023	Lee, JWJ et al. [50]	Bacteria	552	Patients with no neoplasia at colonoscopy	419 TAs only: 321 SSLs only: 62 Both TAs and SSLs: 36	Patients with TAs, SSLs or both at colonoscopy (each group handled separately)	0	Mixed asymptomatic and symptomatic population – individuals ≥ 18 years who were referred for outpatient colonoscopy

Abbreviations: GI, gastrointestinal; HGD, high grade dysplasia; IBD, inflammatory bowel disease; SSL, sessile serrated lesion; TA, tubular adenoma.

### 3.3 Study Methods

The methodology of the studies varied widely.

Definitions of control populations and ‘polyp cases’ were inconsistent (Table 1). Sixteen studies (12 populations) defined controls as those with completely normal colonoscopies [25, 26, 27, 28, 31, 33, 34, 35, 38, 42, 43, 44, 47, 48, 50] or flexible sigmoidoscopies [46]. One study included individuals with no neoplasia as well as those that they considered to have low risk findings (up to two small polyps) [11]. One specifically used patients with haemorrhoids as controls [36]. Four studies [29, 32, 39, 49] used ‘healthy’ individuals where no endoscopy was performed. Of these, one used cohabiting family members [32], one used patients awaiting surgery for non‐gastrointestinal, non‐malignant conditions [49], one used participants recruited in a previous, unrelated study [29] and one used ‘hospitalised’ patients deemed healthy on physical examination [39]. In the remaining four studies the control population was not defined [37, 40, 41, 45]. Studies also differed as to how ‘polyp cases’ were defined. Ten studies included patients with any adenoma [11, 31, 35, 36, 38, 39, 40, 42, 44, 48] and two used patients with ‘high risk’ adenomas only [43, 46]. Two studies included non‐adenomatous polyps (SSLs and hyperplastic), but they were handled differently, with one placing individuals with any polyp type into one heterogeneous group [47], and the other comparing those with only adenomas, those with only SSLs and those with mixed phenotypes to controls and to each other [50].

Reporting of confounding factors (such as use of medications, comorbidities, smoking status etc.) was limited (Table S5). Exclusion criteria were also variable. Whilst many studies excluded individuals with recent antibiotic and/or probiotic use, wide‐ranging timeframes for these exclusions were used, from current use [50] to 6 months prior to recruitment [44].

Protocols were also heterogeneous with regards to the handling of samples, laboratory processes, computational tools, and statistical methods used (Table S6). The sample collection method is known to affect the accuracy and reproducibility of microbiome data [51, 52], however, whilst most studies reported collecting freshly evacuated stool, details of container type and collection method were limited. Only three articles reported the use of DNA/RNA buffers [36, 44, 47] and seven [11, 26, 27, 28, 32, 38, 45] gave no details regarding stool collection. Reporting of storage times (pre and post freezing) and the use of ice, etc., was limited. Where studies did report storage time prior to DNA extraction, there was wide variation—with some studies using archived stool stored for > 15 years [46, 49] and some studies extracting DNA shortly after sample collection [29, 41]. In most studies, sample storage time was not reported. The DNA extraction technique has also been shown to affect microbiome findings [53]. A variety of techniques for this were used across studies. Most studies used a Qiagen kit [25, 26, 27, 28, 29, 32, 34, 35, 36, 37, 38, 41, 44, 46, 50], although the specific kit varied. The remaining studies used kits from other manufacturers [11, 31, 33, 39, 42, 45, 47, 48, 49] or did not report their DNA extraction protocols [43]. All studies used Illumina platforms for sequencing, but read depth was variable. Most studies sequenced around 5Gb of data per sample; however, the specific number of reads was not reported by all studies. Where reported, average high‐quality reads per sample ranged from 1,102,247 ± 643,325 [47] to 44,227,127 [11] (full details can be found in Table S6). All studies reporting bacterial findings to species level (therefore included in data synthesis) used relative rather than absolute abundances; however, a variety of annotation databases were used, including MetaPhlAn [11, 29, 38, 39, 44, 45, 46, 50], Kraken [26, 27, 33, 34, 36, 37, 39, 40] and several others. Although different databases have been shown to produce comparable data [54, 55], this remains a potential confounder.

### 3.4 Quality Appraisal

Newcastle‐Ottawa scores ranged from 1 to 7, out of a possible 9 (Table S4). Only five studies scored > 4; one scoring 7 [46], three scoring 6 [11, 29, 32] and one scoring 5 [42]. The remaining 21 studies should be considered to have high risk of bias.

When considering the individual components of the tool, studies generally scored poorly for ‘Selection’, often because recruitment methods were reported in insufficient detail to establish how participants were selected. In 16 studies [11, 29, 31, 32, 35, 36, 37, 38, 39, 40, 41, 44, 45, 48, 49, 50] the indication for colonoscopy was not reported, meaning that it was unclear whether populations included symptomatic patients as cases and/or controls. ‘Comparability’ scores were generally good, however eight studies [33, 36, 39, 41, 44, 45, 47, 48] scored zero due to not reporting whether case and control groups were age/sex matched or adjusting for these in statistical analysis. ‘Exposure’ scores were fairly consistent, as all studies used histology to identify cases.

### 3.5 Bacterial Findings

#### 3.5.1 Alpha and Beta Diversity

**FIGURE 2:**
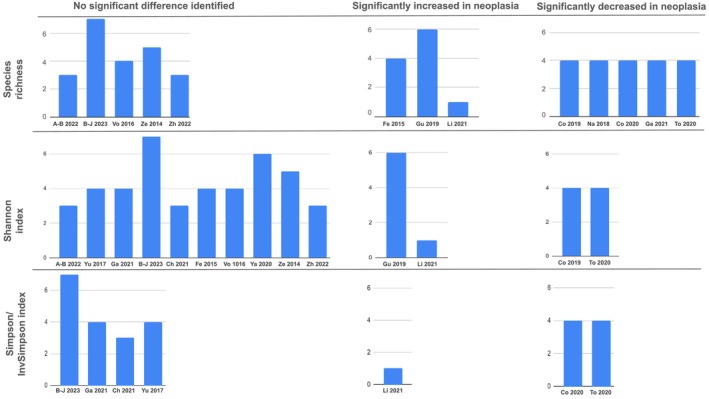
Harvest plot showing bacterial alpha diversity findings across studies (the height of the bar represents the quality appraisal score). A‐B 2022 [47]; B‐J 2023 [46]; Vo 2016 [49]; Ze 2014 [42]; Zh 2022 [39]; Fe 2015 [43]; Gu 2019 [29]; Li 2021 [36]; Co 2019 [27]; Na 2018 [26]; Co 2020 [28]; Ga 2021 [34]; To 2020 [45]; Yu 2017 [25]; Ch 2021 [33]; Ya 2020 [32].

Sixteen studies [25, 26, 27, 28, 29, 32, 33, 34, 36, 39, 42, 43, 45, 46, 47, 49] reported bacterial alpha diversity. All studies used one or more of observed richness, Shannon and Simpson/InvSimpson indices. Richness was reported by 13 studies [26, 27, 28, 29, 34, 36, 39, 42, 43, 45, 46, 47, 49], Shannon index by 14 [25, 27, 29, 32, 33, 34, 36, 39, 42, 43, 45, 46, 47, 49] and Simpson/InvSimpson index by seven [25, 28, 33, 34, 36, 45, 46]. Findings were inconsistent as to whether alpha diversity was increased or decreased in individuals with neoplasia, and was not associated with particularly low or high quality appraisal scores (Figure 2).

Sixteen studies reported bacterial beta diversity, also with inconsistent results. A range of metrics were used, the most common being Bray‐Curtis distance [29, 33, 39, 45, 46, 47], but with some studies using phylogenetic measures, such as UniFrac [31, 37]. There was also variation between the use of weighted and unweighted measures. 12 studies [27, 28, 29, 31, 33, 34, 37, 38, 39, 42, 43] identified distinct clusters discriminating between colorectal neoplasia cases and controls whilst four did not identify any significant differences [36, 45, 46, 47]. This did not appear to be associated with the metric used.

### 3.6 Taxonomy

#### 3.6.1 Bacteria

Seventeen studies [11, 25, 29, 31, 32, 33, 34, 37, 38, 39, 42, 43, 44, 45, 46, 49, 50] reported significantly enriched bacterial species in one or more of the six comparisons (CRC vs. control, control vs. CRC, polyps vs. control, control vs. polyps, CRC vs. polyps, polyps vs. CRC).

Two studies [33, 37] did not report *p* values adjusted for multiple testing or a false discovery rate (FDR); associations reported by these studies have been included based upon their unadjusted *p* values (if < 0.05).

##### 3.6.1.1 Species Enriched in CRC Compared to Controls

Fifteen studies [11, 25, 29, 31, 32, 33, 34, 37, 38, 39, 42, 43, 44, 46, 49] reported bacterial species significantly enriched in individuals with CRC when compared to controls. Across all studies, 332 such species were reported. However, 280 (84%) of these were only identified by a single study and were not reproduced in other populations. Nineteen species were significantly enriched in three or more studies. These are listed in Table 2 in order of the number of studies they were identified in. The most described species were *Fusobacterium nucleatum* (significantly enriched in CRC in 10 studies), *Parvimonas micra* (significantly enriched in nine studies), and *Gemella morbillorum* and *Peptostreptococcus stomatis* (both significantly enriched in seven studies).

**TABLE 2:** Bacterial species enriched in CRC versus controls in ≥ 3 studies, listed in order of most‐to‐least reported (*p* values are reported to three decimal places where available).

Bacterial species	Chang 2021 [33]	Coker 2022 [38]	Feng 2015 [43]	Gao 2020 [31]	Gao 2021 [34]	Gupta 2019 [29]	Tarallo 2019 [44]	Vogtmann 2016 [49]	Yachida 2019 [11]^a^	Yang 2020 [32]	Yang 2021 [37]	Yu 2017 [25]	Zeller 2014 [42]	Zhang 2022 [39]
*Fusobacterium nucleatum* ^b^	*p* = 0.008	BH adj *p* < 0.05		Bonferroni adj *p* < 0.05	BH adj *p* < 0.05			*p* = 0.043	BH adj *p* < 0.05	BH adj *p* < 0.001		BH adj *p* < 0.001	FDR adj *p* < 0.001	BH adj *p* < 0.05
*Parvimonas micra*	*p* = 0.001	BH adj *p* < 0.05	Bonferroni adj *p* < 0.001	Bonferroni adj *p* < 0.05	BH adj *p* < 0.05	FDR adj *p* < 0.001			BH adj *p* < 0.05			BH adj *p* < 0.001		BH adj *p* < 0.05
*Gemella morbillorum*	*p* = 0.005	BH adj *p* < 0.05	Bonferroni adj *p* < 0.001	Bonferroni adj *p* < 0.05					BH adj *p* < 0.05	BH adj *p* < 0.001				BH adj *p* < 0.05
*Peptostreptococcus stomatis*		BH adj *p* < 0.05	Bonferroni adj *p* < 0.001	Bonferroni adj *p* < 0.05		FDR adj *p* < 0.001			BH adj *p* < 0.05				FDR adj *p* = 0.022	BH adj *p* < 0.05
*Prevotella intermedia*		BH adj *p* < 0.05		Bonferroni adj *p* < 0.05	BH adj *p* < 0.05				BH adj *p* < 0.05					BH adj *p* < 0.05
*Solobacterium moorei*		BH adj *p* < 0.05		Bonferroni adj *p* < 0.05			BH adj *p* = 0.036		BH adj *p* < 0.05			BH adj = 0.010		
*Bacteroides fragilis*		BH adj *p* < 0.05			BH adj *p* < 0.05	FDR adj *p* < 0.001								BH adj *p* < 0.05
*Clostridium symbiosum*		BH adj *p* < 0.05	Bonferroni adj *p* < 0.001				BH adj *p* = 0.045							BH adj *p* < 0.05
*Escherichia coli*			Bonferroni adj *p* = 0.001		BH adj *p* < 0.05	FDR adj *p* < 0.001	BH adj *p* = 0.008							
*Filifactor alocis*		BH adj *p* < 0.05		Bonferroni adj *p* < 0.05					BH adj *p* < 0.05	BH adj *p* < 0.001				
*Fusobacterium varium*		BH adj *p* < 0.05			BH adj *p* < 0.05					BH adj *p* = 0.049				BH adj *p* < 0.05
*Parabacteroides distasonis*			Bonferroni adj *p* = 0.002			FDR adj *p* < 0.001					*p* < 0.05 (old onset)			
*Porphyromonas asaccharolytica*		BH adj *p* < 0.05			BH adj *p* < 0.05					BH adj *p* = 0.022			FDR adj *p* = 0.010	
*Clostridium bolteae*		BH adj *p* < 0.05	Bonferroni adj *p* < 0.001											BH adj *p* < 0.05
*Clostridium ramosum*		BH adj *p* < 0.05		Bonferroni adj *p* < 0.05						BH adj *p* < 0.001				
*Flavonifracter plautii*						FDR adj *p* < 0.001					*p* < 0.05 (young onset)			BH adj *p* < 0.05
*Fusobacterium mortiferum*							BH adj *p* = 0.0361	*p* = 0.036	BH adj *p* < 0.05					BH adj *p* < 0.05
*Porphyromonas gingivalis*				Bonferroni adj *p* < 0.05	BH adj *p* < 0.05					BH adj *p* = 0.022				
*Prevotella nigrescens*				Bonferroni adj *p* < 0.05						BH adj *p* = 0.010			FDR adj *p* = 0.022	

^a^ Two studies [11, 42] reported *Fusobacterium nucleatum* by subspecies – for the purposes of this table, these have been combined.

^b^ Yachida et al. report findings by cancer stage – for the purposes of this table, if an organism achieved a *p* value of < 0.05 for any cancer stage this has been reported here (See Table S7 for full results).

##### 3.6.1.2 Species Enriched in Controls Compared to CRC

Sixteen studies [11, 25, 29, 31, 32, 33, 34, 37, 38, 39, 42, 43, 44, 45, 46, 49] reported bacteria which might be considered beneficial, that is, significantly enriched in controls compared to individuals with CRC. Across all studies, 153 of these species were reported. As above, there was limited reproducibility between studies and only 28 species were identified in more than one study. 12 species were significantly enriched in controls compared to CRC in three or more studies (Table 3).

**TABLE 3:** Bacterial species enriched in controls versus CRC in ≥ 3 studies, listed in order of most‐to‐least reported (*p* values reported to three decimal places where available).

Bacterial species	Chang 2021 [33]	Coker 2022 [38]	Gao 2020 [31]	Gao 2021 [34]	Gupta 2019 [29]	Tarallo 2019 [44]	Touchefeu 2020 [45]	Yang 2020 [32]	Yang 2021 [37]	Zeller 2014 [42]	Zhang 2022 [39]
*Faecalibacterium prausnitzii*	*p* = 0.005		Bonferroni adj *p* < 0.05	BH adj *p* < 0.05	FDR adj *p* = 0.019	BH adj *p* = 0.043	BH adj *p* = 0.014		*p* < 0.05 (old onset)		BH adj *p* < 0.05
*Eubacterium eligens*	*p* = 0.006		Bonferroni adj *p* < 0.05	BH adj *p* < 0.05		BH adj *p* = 0.005				FDR adj *p* = 0.021	BH adj *p* < 0.05
*Anaerostipes hadrus*	*p* = 0.002	BH adj *p* < 0.05	Bonferroni adj *p* < 0.05	BH adj *p* < 0.05					*p* < 0.05 (old onset)		
*Eubacterium rectale*			Bonferroni adj *p* < 0.05	BH adj *p* < 0.05	FDR adj *p* < 0.001				*p* < 0.05 (young and old onset)		BH adj *p* < 0.05
*Bifidobacterium adolescentis*				BH adj *p* < 0.05	FDR adj *p* < 0.001	BH adj *p* = 0.039					BH adj *p* < 0.05
*Roseburia intestinalis*		BH adj *p* < 0.05	Bonferroni adj *p* < 0.05						*p* < 0.05 (young and old onset)		BH adj *p* < 0.05
*Roseburia inulinivorans*		BH adj *p* < 0.05	Bonferroni adj *p* < 0.05					BH adj *p* = 0.006			BH adj *p* < 0.05
*Eubacterium hallii*		BH adj *p* < 0.05		BH adj *p* < 0.05						FDR adj *p* = 0.018	
*Lactobacillus ruminis*				BH adj *p* < 0.05	FDR adj *p* < 0.001						BH adj *p* < 0.05
*Megamonas hypermegale*		BH adj *p* < 0.05		BH adj *p* < 0.05							BH adj *p* < 0.05
*Roseburia hominis*			Bonferroni adj *p* < 0.05	BH adj *p* < 0.05					*p* < 0.05 (old onset)		
*Streptococcus salivarius*			Bonferroni adj *p* < 0.05	BH adj *p* < 0.05						FDR adj *p* = 0.017	

##### 3.6.1.3 Species Enriched in Polyps Compared to Controls

Six studies [11, 31, 39, 43, 46, 50] described significantly enriched bacteria in individuals with polyps when compared to controls. This resulted in > 100 species being reported; no species were demonstrated in more than one study.

Only one study [50] separated and compared different polyp types and here the authors identified significant organisms associated with sessile serrated lesions (SSLs) and adenomas when compared to controls and to each other.

#### 3.6.2 Species Enriched in Controls Compared to Polyps

When controls were compared to individuals with polyps, six studies [11, 31, 39, 43, 44, 50] also identified bacterial species that might be considered beneficial. Only one species out of the > 100 reported was identified by more than one study: *Eubacterium xylanophilum* (identified in two studies with one reporting an unadjusted *p* = 0.003 [44] and the other a Benjamini‐Hochberg adjusted *p* < 0.05 [39]).

##### 3.6.2.1 Species Enriched in CRC Compared to Polyps

When patients with CRC were compared to those with polyps, six studies [31, 34, 38, 39, 42, 43] reported significantly enriched bacteria associated with CRC. A total of 78 species were reported across these studies. 12 of these were identified by more than study, the most reported being *Parvimonas micra* which was identified in four studies (Table 4).

**TABLE 4:** Bacterial species enriched in CRC versus polyps in more than one study.

Bacterial species	Coker 2022 [38]	Gao 2020 [31]	Gao 2021 [34]	Feng 2015 [43]	Zeller 2014 [42]	Zhang 2022 [39]
*Parvimonas micra*	BH adj *p* < 0.05			Bonferroni adj *p* = 0.0002	FDR adj *p* = 0.0232	BH adj *p* < 0.05
*Clostridium symbiosum*	BH adj *p* < 0.05			Bonferroni adj *p* = 0.0000		BH adj *p* < 0.05
*Fusobacterium nucleatum*	BH adj *p* < 0.05		BH adj *p* < 0.05			BH adj *p* < 0.05
*Fusobacterium sp oral taxon 370*		Bonferroni adj *p* < 0.05		Bonferroni adj *p* = 0.0002		BH adj *p* < 0.05
*Gemella morbillorum*	BH adj *p* < 0.05			Bonferroni adj *p* = 0.0001		BH adj *p* < 0.05
*Peptostreptococcus stomatis*	BH adj *p* < 0.05			Bonferroni adj *p* = 0.0091	FDR adj *p* = 000266	
*Bilophila wadsworthia*				Bonferroni adj *p* = 0.0016		BH adj *p* < 0.05
*Clostridium asparagiforme*				Bonferroni adj *p* = 0.0085		BH adj *p* < 0.05
*Clostridium ramosum*	BH adj *p* < 0.05	Bonferroni adj *p* < 0.05				
*Dialister invisus*	BH adj *p* < 0.05			Bonferroni adj *p* = 0.0277		
*Odoribacter splanchnicus*				Bonferroni adj *p* = 0.0008		BH adj *p* < 0.05
*Prevotella intermedia*	BH adj *p* < 0.05					BH adj *p* < 0.05

##### 3.6.2.2 Species Enriched in Polyps Compared to CRC

Conversely, five studies [34, 38, 39, 42, 43] reported significantly enriched bacteria in individuals with polyps compared to those with CRC (total of 26 species). Only two of these were identified by more than study. *Eubacterium rectale* was identified in three studies [34, 38, 42] (adjusted *p* values of < 0.05, < 0.05 and 0.023 respectively) and *Blautia hansenii* in two studies [34, 39] (both reporting adjusted *p* values of < 0.05).

### 3.7 Viruses, Fungi and Archaea

Few studies reported non‐bacterial findings—four reported viruses [26, 34, 39, 48], two reported fungi [27, 35] and one reported archaea [28].

Of the four studies reporting findings on viruses, three found no significant differences in alpha diversity [34, 39, 48] whereas one reported bacteriophage richness to be increased in CRC compared to controls [26]. For beta diversity, two studies reported distinct clusters discriminating cases and controls [34, 39] and one found no significant difference [48]. Across all four studies, 93 viral species were found to be significantly enriched in patients with CRC compared to controls, but there was no agreement between studies and no single species was reproduced. Similarly, 53 species were enriched in controls compared to CRC, but again there was no agreement between studies. Zhang et al. [39] were the only authors to study viral findings in patients with polyps and reported three species enriched in individuals with polyps compared to controls, 21 species enriched in controls compared to polyps and 16 species enriched in CRC compared to polyps.

Neither of the studies reporting mycobiome findings [27, 35] identified significant differences in fungal richness or Shannon index. One reported a difference in beta diversity between cases and controls [35] with the other finding no significant difference [27]. In contrast with viruses, fungal findings demonstrated some reproducibility. Six species were significantly enriched in CRC compared to controls in both studies (Table 5) and one species was identified by both papers as enriched in controls compared to CRC (*Exophiala mesophile*, with adjusted *p* values of 0.009 [27] and < 0.05 [35]).

**TABLE 5:** Fungal species enriched in CRC compared to controls.

Fungal species	Coker 2019 [27]	Gao 2022 [35]
*Pseudogymnoascus sp VKM F‐4520 (FW‐2644)*	FDR adj *p* = 0.0001	FDR adj *p* < 0.01
*Debaryomyces fabryi*	FDR adj *p* = 0.2087	FDR adj *p* < 0.05
*Aspergillus versicolor*	FDR adj *p* = 0.0167	FDR adj *p* < 0.01
*Aspergillus rambellii*	FDR adj *p* = 0.006	FDR adj *p* < 0.01
*Aspergillus ochraceoroseus*	FDR adj *p* = 0.0388	FDR adj *p* < 0.05
*Aspergillus flavus*	FDR adj *p* = 0.0332	FDR adj *p* < 0.01

In the single study which reported archaea findings [28], no significant difference in either richness or InvSimpson index was identified; however, distinct clusters discriminating between cases and controls (as well as between adenoma, early cancer and late cancer) were reported for beta diversity. Nine species were enriched in CRC and 19 were depleted (FDR adjusted *p* values < 0.05).

## 4 Discussion

In this first systematic review focusing specifically on studies using modern shotgun sequencing in stool samples, 26 articles reporting findings from 22 patient populations were identified. It demonstrated some microbial signals for colorectal neoplasia that were reproduced across different populations. However, there was considerable variation in study quality with most of the included studies judged to be high risk of bias. Consistent with previous reviews (which did not limit consideration to shotgun sequencing studies), sample sizes were generally small and there was extensive heterogeneity in study methodology, including but not limited to: inclusion and exclusion criteria, definitions of control populations, definitions of ‘polyp cases’, handling of stool samples, laboratory protocols, computational tools and statistical analysis. This is, in part, due to variation in study type and aim (e.g., some studies having a biology‐driven, discovery focus compared to others with a more clinical, translational approach), and means that direct comparisons between these studies should not be made. Researchers should exercise caution when interpreting findings, particularly if attempting to pool data.

Whilst cases for CRC were well defined and histologically proven in all studies, polyp cases and controls were heterogeneous. There is a lack of consensus on polyp classifications and definitions internationally, with British [56], European [57] and American [58] colonoscopy surveillance guidelines all using slightly different classifications. Similarly, agreement on the most appropriate controls must be reached as these were also variable. Whilst individuals who have been endoscopically proven to have no neoplasia are often used, these patients have usually been referred for colonoscopy due to GI symptoms and/or positive FIT, therefore may not be truly ‘healthy’ or representative of the population from which the cases arose.

As well as participant selection, there was also heterogeneity in other methodological factors with the potential to affect microbiome findings. Study protocols may affect results due to their influence on DNA quality (sampling handling and DNA extraction), level of detail (read depth) and species identification (annotation database).

Confounding factors related to populations (geography, diet, comorbidities, medication, etc.), samples (collection method, storage times/conditions and laboratory processes) and analysis (computational tools and statistical methods) are poorly controlled for in the existing literature and future studies must address this. This degree of heterogeneity is the major limitation of the current body of evidence and, as previously reported [9] means that meta‐analysis and large‐scale pooling of data cannot be performed robustly. Whilst it is essential that data from large, diverse populations are recruited and pooled, these groups will have many such confounders, which must be minimised or adjusted for in statistical analysis. As discussed in our previously published commentary [59], the identification of reproducible organisms (and subsequent development of clinically useful biomarkers) is unlikely to be achieved unless these issues are addressed and standardised study protocols have been agreed by international consensus to minimise this heterogeneity in future research.

Whilst these issues mean that no definite conclusions can be drawn from the current body of evidence, this review has identified some potentially interesting findings which should guide future research questions. Many of the species most consistently associated with CRC were those more commonly seen in the oral cavity than the colon, an association that has been widely reported [60, 61] and for which several mechanistic hypotheses have been proposed. Colonic lesions have been found to involve polymicrobial biofilms containing oral bacteria (such as *Fusobacterium nucleatum* and others), with such biofilms being a common feature of oral diseases such as periodontitis [60, 62]. Other mechanisms related to the virulence mechanisms of oral bacteria and their potential ability to inhibit apoptosis and/or modulate colonic inflammatory and immune responses have been suggested [60]. Oral bacteria *Fusobacterium nucleatum* and *Parvimonas micra* were the most consistently reported species (identified in 10 and nine studies respectively). *Fusobacterium nucleatum* was also one of the key organisms identified by the previous systematic review by Yu et al. [9] Both species were identified in populations from Europe and Asia, suggesting that these could be signatures which are seen across different ethnicities and geographical locations. These organisms were reported by studies with a range of Newcastle‐Ottawa scores, and there were no other trends identified, such as a specific methodology, leading to them being more likely to be reported. Yachida et al. [11] compared patients with CRC at different stages and reported that abundance of *Fusobacterium nucleatum* increased sequentially from intramucosal carcinoma to more advanced stages (*p* < 0.005). Interestingly, this study also found that the abundance of species associated with CRC decreased after patients had curative surgery. Both *Fusobacterium nucleatum* and *Parvimonas micra* were also found to be enriched in CRC patients compared to those solely with polyps (in three and four studies for *Fusobacterium nucleatum* and *Parvimonas micra* respectively), suggesting that they could act as differentiators between patients with cancer and those with polyps. These findings support the suggestion that some microbiome alterations occur as a continuum as the adenoma‐carcinoma sequence progresses and that a ‘driver‐passenger’ [63] process occurs.

*Escherichia coli* was identified by four studies as being significantly enriched in CRC patients compared to controls. A subspecies of this bacteria carrying the polyketide synthetase (*pks*) island (*pks* ^+^ *E. coli* ) has been widely reported to be associated with CRC [64, 65] and in gnotobiotic mouse models it has been shown to contribute to CRC tumorigenesis [66]. The mechanism is thought to be due to its production of colibactin which alkylates DNA inducing interstrand crosslinks and double‐strand breaks in epithelial cell lines [64]. *pks* ^+^ *E. coli* , along with other potentially pathogenic colonic bacteria, such as toxin‐forming *Bacteroides fragilis* (significantly enriched in three studies) have been reported as potential ‘driver’ organisms, producing DNA damaging compounds, in comparison to the ‘passengers’ (including *Fusobacterium nucleatum* and other oral bacteria), which are able to colonise the tumour microenvironment and promote tumorigenesis [67].

There was a wide range of less commonly identified organisms, with a total of 332 bacterial species identified as being enriched in CRC compared to controls across all studies. The overwhelming majority of these were only identified by a single study and not reproduced in other populations. As discussed, this could be due to the heterogeneity of the study populations/methods and uncontrolled confounding.

There were less data available for colorectal polyps, with only 14 studies including individuals with polyps. There was even less reproducibility of species associated with polyps than with CRC. In the only study [50] to compare different histological polyp types, significant differences were identified. Whilst it may be that polyps have less strong signals than CRC, it could also be that when heterogeneous patient groups are used as ‘polyp cases’ associations become more difficult to detect.

Another limitation of the current body of evidence is that all studies were cross‐sectional, using samples collected at a single time point. Longitudinal studies with prospectively collected samples are required to address questions about causality and how the microbiome changes over time. This is essential to answer the fundamental question of whether dysbiosis leads to neoplasia or the lesions themselves alter the microbiome (or both, as in the driver‐passenger model).

This systematic review was designed and performed using robust methodology, minimising bias. The broad search strategy resulted in a large number of titles and abstracts being screened, reducing the likelihood of missing any relevant studies. However, one limitation is that for practical purposes we excluded articles not written in English, meaning that there is the possibility that some relevant findings could have been missed. A further limitation is that, given that there are currently no accepted guidelines for the reporting of data in systematic reviews of shotgun sequencing studies, for pragmatic purposes, we chose to only include species in data synthesis if they were found to be ‘significantly’ enriched (adjusted *p* value < 0.05) in more than one study. In comparisons between CRC and controls this was increased to more than two studies due to the large number of reported associations. Therefore, it is possible that relevant species have not been included if they were only identified by one or two studies. Similarly, we included findings from two studies where *p* values were not adjusted for multiple testing. Whilst this could introduce bias, we made the judgement to include these two studies in order to be comprehensive, recognising the risk of losing important data if they were excluded. This is a further example of the limited comparability between the studies in this review, a major limitation of the current evidence base.

Additionally, there is no accepted quality appraisal tool specifically designed for assessing the risk of bias or methodological quality in shotgun sequencing studies. We chose to use an existing tool (the Newcastle‐Ottawa scale (NOS)) as it is well known and widely used. However, it is important to note that the NOS is designed for population‐based or clinical case–control studies so this may not be the optimal tool for assessing the quality of some studies in this review. Many studies were judged as poor quality due to their lack of stringent clinical protocols; however often these studies had a biology/bioinformatic (rather than clinical) focus and included some of the important, early discovery data in this field [43, 49]. Consensus protocols for systematic reviews of shotgun sequencing studies, including a specifically designed quality appraisal tool, are important to advance this research field.

Given the nature of metagenomic data, these studies do not report an effect estimate which could be combined in a quantitative meta‐analysis. In order to perform an individual participant data meta‐analysis, the raw sequencing data from each study would be required. Whilst this is publicly available for some studies, it is not available for all those that were included and was beyond the scope of this review. A meta‐analysis of this type would be a significant undertaking but would be an extremely valuable contribution to the field.

While the evidence‐base in this area has evolved rapidly over the past decade, we have only identified one study [68] that meets our inclusion criteria, which has been published since the date of our searches. This small Spanish study (30 CRC cases, 30 polyp patients, 30 controls) identified some significant associations with specific taxa but reported results only to genus level, so would not have been included in our synthesis. Nor does it challenge our conclusions.

In summary, this is the first systematic review solely focusing on shotgun sequencing studies using faecal samples. Whilst reproducible signals have been identified, the quality of evidence limits the real‐world implications of these findings. If robust, global data could be achieved in future, it may be possible to draw firmer conclusions about specific organisms. This may mean that microbiome‐based screening or risk stratification tools could be developed, and future research could focus on assessing the predictive performance of these in real‐world populations. Currently, biomarkers such as FIT are widely used to risk stratify the need for colonoscopy, yet the number of false positives is high. If the addition of microbial markers improved predictive performance, then this would have important implications for clinical practice, potentially reducing the number of unnecessary colonoscopies performed, reducing risk to patients and burden on health care systems.

Contributions are described below using CRediT [69] taxonomy.

## Author Contributions

**Sarah Manning:** conceptualization, formal analysis, investigation, methodology, project administration, validation, writing – original draft, writing – review and editing. **Eleanor Hackney:** investigation, validation, writing – review and editing. **Yashvee Dunneram:** investigation, validation, writing – review and editing. **Mark A. Hull:** funding acquisition, supervision, writing – review and editing. **Suparna Mitra:** investigation, methodology, validation, writing – review and editing. **Christopher J. Stewart:** supervision, writing – review and editing. **Panayiotis Louca:** supervision, validation, writing – review and editing. **Nick Meader:** methodology, writing – review and editing. **Linda Sharp:** conceptualization, funding acquisition, methodology, supervision, visualization, writing – review and editing. **Colin Rees:** conceptualization, funding acquisition, methodology, supervision, visualization, writing – review and editing.

## Conflicts of Interest

The authors report no conflicts of interest related to this systematic review. Of note, as part of COLO‐COHORT [70], members of the review team are involved in original research studying the associations between the faecal microbiome and colorectal neoplasia.

## Supporting information


**Data S1:** apt70252‐sup‐0001‐Appendices.docx.

## Data Availability

The data that support the findings of this study are available from the corresponding author upon reasonable request.
