# Supplementary material for: Systematic Review: The Relationship Between the Faecal Microbiome and Colorectal Neoplasia in Shotgun Metagenomic Studies
Source: Aliment Pharmacol Ther. 2025 Aug 12;62(6):568–84. doi: 10.1111/apt.70252 (PMC12395896; doi:10.1111/apt.70252)
Supplement: Supplementary file 1 — Data S1: apt70252‐sup‐0001‐Appendices.docx. [file APT-62-568-s001.docx]

Supplementary Material

Appendix 1: Supplementary methods

Eligibility criteria

The eligibility criteria were developed using the PICO (Population, Intervention, Comparator, Outcome) framework:

- Population: Humans with colorectal neoplasia
- Intervention/Exposure: None
- Comparator: Non-neoplasia controls
- Outcome: Faecal microbiome findings (alpha diversity, beta diversity and taxonomy)

Based on the PICO, the following eligibility criteria was developed:

1. Articles must be an original study where the stool of humans with colorectal neoplasia (defined as CRC or adenomatous, serrated and hyperplastic polyps) and non-neoplasia controls was analysed using whole metagenome shotgun sequencing
2. Studies must report findings comparing the composition (diversity and taxonomy) of the faecal microbiome of cases (participants with colorectal neoplasia) to that of controls (no neoplasia).

As discussed in the main text, patients with neoplasia associated with IBD or genetic conditions were excluded. Papers which included these as well as the population of interest were considered eligible for inclusion only if the data were presented separately. If studies reported participants with colorectal neoplasia but did not mention these excluded conditions, they were assumed to be sporadic neoplasia populations and were eligible for inclusion.

Studies which assessed the effect of an intervention with the potential to influence the microbiome (such as cancer treatment, probiotics, FMT, etc.) were included if pre-intervention data were presented separately. These data were included in data synthesis, but post-intervention data were not.

One study^11^ included both neoplasia-free controls and individuals with what they considered low risk findings (up to two small polyps) in their control population. This was discussed by the reviewers, and a decision was made to include this study due to its large sample size so that important findings were not missed.

Reviews and meta-analyses were excluded. Articles which reported post-hoc analysis of previously published data were not included (but backwards citation searching was performed to ensure that the original dataset was included). Animal or cell studies were excluded. Conference abstracts were excluded due to insufficient detail in the findings. Articles not written in English were excluded for practical reasons and this has been acknowledged as a limitation of this review.

Data sources

The search strategies for MEDLINE and EMBASE (both via OVID) can be seen in Supplementary Tables 1 & 2. Backwards citation searching was performed from the reference lists of included articles, potentially relevant review articles and those reporting previously published datasets.

*Supplementary Table 1: Search strategy (MEDLINE)*

| 1. | colorectal.mp. or exp Colorectal Neoplasms/ |
| --- | --- |
| 2. | exp Intestine, Large/ |
| 3. | rectum.mp. |
| 4. | rectal.mp. |
| 5. | colon*.mp. |
| 6. | bowel.mp. |
| 7. | exp Neoplasms/ or neoplas*.mp. |
| 8. | cancer.mp. |
| 9. | carcinoma.mp. |
| 10. | exp Adenocarcinoma/ or adenocarcinoma.mp. |
| 11. | exp Intestinal Polyps/ |
| 12. | polyp*.mp. |
| 13. | adenoma*.mp. |
| 14. | serrated.mp. |
| 15. | hyperplastic.mp. |
| 16. | microbiome.mp. |
| 17. | exp Microbiota/ or microbiota.mp. |
| 18. | dysbiosis.mp. or exp Dysbiosis/ |
| 19. | 1 or 2 or 3 or 4 or 5 or 6 |
| 20. | 7 or 8 or 9 or 10 or 11 or 12 or 13 or 14 or 15 |
| 21. | 16 or 17 or 18 |

*Supplementary Table 2: Search strategy (EMBASE)*

| 1. | large intestine.mp. or exp large intestine/ |
| --- | --- |
| 2. | rectum.mp. |
| 3. | rectal.mp. |
| 4. | colon*.mp. |
| 5. | bowel.mp. |
| 6. | exp Colorectal Neoplasms/ or colorectal neoplas*.mp. |
| 7. | cancer.mp. |
| 8. | adenocarcinoma.mp. |
| 9. | polyp*.mp. or exp colorectal polyp/ |
| 10. | adenoma*.mp. |
| 11. | serrated.mp. |
| 12. | hyperplastic.mp. |
| 13. | exp microbiome/ or microbiome.mp. |
| 14. | microbiota.mp. |
| 15. | dysbiosis.mp. or exp dysbiosis/ |
| 16. | 1 or 2 or 3 or 4 or 5 |
| 17. | 6 or 7 or 8 or 9 or 10 or 11 or 12 |
| 18. | 13 or 14 or 15 |
| 19. | 16 and 17 and 18 |

Data extraction

Supplementary Table 3 shows the details of the data extraction form.

*Supplementary Table 3: Data extracted from articles*

| Publication information | Title  Authors  Journal  Year of publication |
| --- | --- |
| Study information | Study dates  Study location(s)  Study design  Inclusion criteria  Exclusion criteria |
| Methods | Source of cases  Source of controls  Stool sampling method  Stool sampling timing relative to colonoscopy and bowel preparation  Sample storage method  Sample storage time  DNA extraction technique  Library preparation technique  Sequencing platform  Read lengths  Sequencing depth  Annotation database used |
| Sample size | Number of CRC cases  Number of polyp cases  Number of controls |
| Participant information | Ethnicity  Sex  Age  BMI  Smoking status  Comorbidities  Antibiotic use  Probiotic use  Indication for colonoscopy |
| Clinical findings | Location of neoplasia  For polyp cases:   - Mean number of polyps - Histological types (adenomatous, serrated, and hyperplastic polyps)   Other significant colonoscopy findings |
| Microbial findings | Difference in alpha diversity between cases and controls (richness, Shannon and Simpson indices)  Difference in beta diversity between cases and controls  Significantly enriched† organisms in:   - CRC vs controls - Controls vs CRC - Polyps vs controls - Controls vs polyps - CRC vs polyps - Polyps vs CRC |
| Interpretation of findings | Authors key conclusions  Any additional information |
| Limitations | Limitations reported by authors  Conflicts of interests declared |

†Species were considered significantly enriched if they were found to have an adjusted p value or FDR of <0.05. If studies did not report adjusting for multiple comparisons, an unadjusted p value <0.05 was accepted due to the potential for loss of important data if they were excluded.

Appendix 2: Supplementary results

*Supplementary Table 4: Newcastle-Ottawa scores for each study*

| **Study** | **Selection score (max.4)** | **Comparability score (max.2)** | **Exposure score (max. 3)** | **Total score** |
| --- | --- | --- | --- | --- |
| Bucher-Johannessen 2023^46^ | 3 | 2 | 2 | 7 |
| Gupta 2019^29^ | 3 | 2 | 1 | 6 |
| Yachida 2019^11^ | 2 | 2 | 2 | 6 |
| Yang 2020^32^ | 2 | 2 | 2 | 6 |
| Zeller 2014^42^ | 2 | 2 | 1 | 5 |
| Coker 2019^27^ | 1 | 2 | 2 | 4 |
| Coker 2020^28^ | 1 | 2 | 2 | 4 |
| Coker 2022^38^ | 1 | 2 | 2 | 4 |
| Feng 2015^43^ | 1 | 2 | 2 | 4 |
| Gao 2020^31^ | 1 | 2 | 2 | 4 |
| Gao 2021^34^ | 1 | 2 | 2 | 4 |
| Gao 2022^35^ | 1 | 2 | 2 | 4 |
| Hannigan 2018^48^ | 2 | 0 | 2 | 4 |
| Lee 2023^50^ | 1 | 2 | 2 | 4 |
| Lv 2023^40^ | 2 | 0 | 2 | 4 |
| Nakatsu 2018^26^ | 1 | 2 | 2 | 4 |
| Tarallo 2019^44^ | 2 | 0 | 2 | 4 |
| Touchefeu 2020^45^ | 2 | 0 | 2 | 4 |
| Vogtmann 2016^49^ | 1 | 2 | 1 | 4 |
| Yang 2021^37^ | 1 | 2 | 2 | 4 |
| Yu 2017^25^ | 2 | 1 | 1 | 4 |
| Avelar-Barragan 2022^47^ | 1 | 0 | 2 | 3 |
| Chang 2021^33^ | 1 | 0 | 2 | 3 |
| Zhang 2022^39^ | 2 | 0 | 1 | 3 |
| Zhang 2023^41^ | 1 | 0 | 2 | 3 |
| Liu 2021^36^ | 0 | 0 | 1 | 1 |

*Supplementar*y *Table 5: Demographic details and exclusion criteria for each study*

| **Study** | **Ethnicity** | **Sex** | **Age** | **BMI** | **Smoking status** | **Comorbidities** | **Location of neoplasia** | **Exclusion criteria** |
| --- | --- | --- | --- | --- | --- | --- | --- | --- |
| Avelar-Barragan 2022^47^ | White – 58%  Black – 1%  Asian – 16%  Hispanic – 11% Other/Unknown – 14% | Male – 48%  Female – 39%  Other/Unknown – 13%    (data for cases and controls not given separately) | Median - 65    (data for cases and controls not given separately) | Median – 26    (data for cases and controls not given separately) | Not reported | Not reported | Not reported | - Pregnancy - IBD - Antibiotics 6 weeks prior to colonoscopy |
| Bucher-Johannessen 2023^46^ | All Norwegians (race not reported) | **Controls:**  Male – 68.2%  Female – 31.8%  **HRA:**  Male – 66.7%  Female – 33.3%  **CRC:**  Male – 28.6%  Female – 71.4% | **Controls:**  Median – 57  Range – 54-64  **HRA:**  Median – 58  Range – 53-64  **CRC:**  Median – 61  Range – 55-66 | Not reported | Not reported | Not reported | Not reported | - Previous open colorectal surgery - Need for long-lasting attention and nursing services - On-going cytotoxic treatment/radiotherapy for malignant disease - Severe chronic cardiac or lung disease - Heart valve replacement on lifelong anticoagulant therapy - A coronary event or CVA during the last 3 months - Resident abroad |
| Chang 2021^33^ | All Chinese | **Cases:**  Male – 100%  Female – 0%    (No details reported for controls) | **Cases:**  Range – 38-77    (No details reported for controls) | **Cases:**  Mean – 21.25  SD – 1.64    (No details reported for controls) | Not reported | Not reported | Not reported | - Pre-existing disease - No antibiotics or probiotics for three months prior |
| Coker 2019^27^ | All from Hong Kong, no further detail reported | **Controls:**  Male – 55.4%  Female – 44.6%    **Cases:**  Male – 64.4%  Female – 35.6% | **Controls:**  Mean – 58.51  SD – 7.55    **Cases:**  Mean – 65.90  SD – 10.61 | **Controls:**  Mean – 23.87  SD – 3.31    **Cases:**  Mean – 24.07  SD – 3.1 | Not reported | Not reported | Not reported | As per Yu 2017 |
| Coker 2020^8^ | As per Coker 2019 | As per Coker 2019 | As per Coker 2019 | As per Coker 2019 | Not reported | Not reported | Not reported | As per Yu 2017 |
| Coker 2022^38^ | All from Hong Kong, no further detail reported | **Controls:**  Male – 46.1%  Female – 53.9%  **Adenoma:**  Male – 58.6%  Female – 41.4%  **CRC:**  Male – 54.2%  Female – 45.8% | **Controls:**  Mean – 64.03  SD – 6.84    **Adenoma:**  Mean – 65.84  SD – 5.53    **CRC:**  Mean – 73.21  SD – 10.37 | **Controls:**  Obese – 12.5%  Not obese – 87.5%  **Adenoma:**  Obese – 21.4%  Not obese – 78.6%  **CRC:**  Obese – 13.6%  Not obese – 86.4% | Not reported | Not reported | Not reported | None reported |
| Feng 2015^43^ | All Caucasians | **Controls:**  Male – 57.9%  Female – 42.1%  **Advanced adenoma:**  Male – 50%  Female – 50%  **CRC:**  Male – 60.9%  Female – 39.1% | **Controls:**  Mean – 67.0  **Advanced adenoma:**  Mean – 66.5  **CRC:**  Mean – 67.1 | **Controls:**  Mean – 27.6  **Advanced adenoma:**  Mean – 28.0    **CRC:**  Mean – 26.5 | Current smokers – 12%  Ex/never smoked – 88% | **Controls:**  Diabetes – 20.6%  Advanced adenoma:  Diabetes – 10.6%  **CRC:**  Diabetes – 23.9% | **Advanced adenoma**:  Right – 31.8%  Left – 34.1%  Rectum – 34.1%    **CRC:**  Right – 17.4%  Left – 23.9%  Rectum –58.7% | None reported |
| Gao 2020^31^ | All from China – no further detail reported | **Controls:**  Male – 30%  Female – 70%  **Polyps:**  Male – 62%  Female – 38%  **Cancer:**  Male – 60%  Female – 40% | **Controls:**  Mean – 64.96  SD – 10.44  **Polyps:**  Mean – 63.07  SD – 12.84  **Cancer:**  Mean – 65.79  SD – 12.73 | **Controls:**  Mean – 23.45  SD – 2.35  **Polyps:**  Mean – 23.13  SD – 2.91  **Cancer:**  Mean – 23.23  SD – 3.4 | Not reported | Not reported | **CRC:**  Ascending – 30%  Transverse – 7%  Descending – 10% Sigmoid – 33%  Rectum – 70%  Undefined – 5%  (Location of polyps not reported) | All participants:   - Exposure to antibiotics within previous one month - Previous radio/ chemotherapy for cancer   Healthy controls only:   - Any cancer history - PPI, probiotics, prebiotics or synbiotics in proceeding month - Previous surgery on digestive tract - History of chronic digestive tract diseases |
| Gao 2021^34^ | All from China – no further detail reported | **Controls:**  Male – 42%  Female – 58%    **Adenoma:**  Male – 54%  Female – 46%    **Cancer:**  Male – 59%  Female – 41% | **Controls:**  Mean – 60.23  SD – 5.06  **Adenoma:**  Mean – 63.22  SD – 7.29  **Cancer:**  Mean – 61.85  SD – 10.96 | **Controls:** Mean – 23.32  SD – 1.97    **Adenoma:**  Mean –23.81  SD – 3.03    **Cancer:**  Mean – 22.96  SD – 3.85 | Not reported | Not reported | **CRC:**  Ascending – 25.4% Transverse – 7.9% Descending – 3.2%  Sigmoid – 12.7% Rectum – 63.5%  (Location of polyps not reported) | - Exposure to antibiotics, probiotics or prebiotics within one month - A history of gastrointestinal - Diagnosed with acute or chronic diarrhoea and hepatitis |
| Gao 2022^35^ | As per Gao 2021 | As per Gao 2021 | As per Gao 2021 | As per Gao 2021 | Not reported | Not reported | As per Gao 2021 | As per Gao 2021 |
| Gupta 2019^29^ | All Indian | **Controls:**  Male – 36.7%  Female – 63.3%  **Cases:**  Male – 62.1%  Female – 37.9% | **Controls:**  Mean – 41.4  **Cases:**  Mean – 59.8 | **Controls:**  Mean – 22.8  **CRC:**  Mean – 20.2 | Not reported | 'Serious medical conditions' excluded | Rectum – 50%  Sigmoid – 20%  Right colon – 30% | - Any previously diagnosed serious medical conditions - Recent use of antibiotics - Incomplete medical information |
| Hannigan 2018^48^ | **Controls:**  Non-Hispanic White – 70% Other – 30%  **Adenoma:** Non-Hispanic White – 90% Other – 10%  **Cancer:** Non-Hispanic White – 93% Other – 7% | **Controls:**  Male – 36.7%  Female – 63.3%  **Adenoma:**  Male – 60.0%  Female – 40.0%  **CRC:**  Male – 70.0%  Female – 30.0% | **Controls:**  Mean – 55.3  SD – 9.2  **Adenoma:**  Mean – 61.3  SD – 11.1  **CRC:**  Mean – 59.4  SD – 11.0 | **Controls:**  Mean – 26.6  SD – 5.2  **Adenoma:**  Mean – 27.4  SD – 4.4  **CRC:**  Mean – 30.7  SD – 7.2 | Not reported | Not reported | Not reported | Not reported |
| Lee 2023^50^ | 'Mostly white' – no other details reported | **Controls:**  Male – 43.5%  Female – 56.5%  **TA only:**  Male – 60.4%  Female – 39.6%  **SSA only:**  Male – 43.5%  Female – 56.5%  **Both TA and SSA:**  Male – 63.9%  Female – 36.1% | **Controls:**  Mean – 59.4  SD – 10.0  **TA only:**  Mean – 64.0  SD – 9.6    **SSA only:**  Mean – 57.8  SD – 11.1  **Both TA and SSA:**  Mean – 64.3  SD – 9.0 | **Controls:**  Mean – 27.0  SD – 5.5  **TA only:**  Mean – 27.8  SD – 5.7    **SSA only:**  Mean – 27.0  SD – 5.8    **Both TA and SSA:**  Mean – 27.0  SD – 4.1 | **Controls:**  Ever smoked –30.7% Never smoked – 69.3%      **TA only:**  Ever smoked – 41.5%  Never smoked – 58.5%    **SSA only:**  Ever smoked –  33.9%  Never smoked –66.1%  **Both TA and SSA:**  Ever smoked – 33.3%  Never smoked – 66.7% | **Controls:**  Cardiovascular disease **–** 3.8%  **TA only:**  Cardiovascular disease **–** 7.5%  **SSA only:**  Cardiovascular disease **–** 1.6%  **Both TA and SSA:**  Cardiovascular disease **–** 13.9% | **TA only:** Left – 15.6% Right – 63.2% Pan-colonic – 21.2%  **SSA only:** Left – 12.9% Right – 72.6% Pan-colonic – 14.5%  **TA and SSA:**  Left – 0% Right – 80.6% Pan-colonic – 19.4% | - Unable to provide stool samples - On concomitant antibiotics or probiotic - Diagnosed with colitis - Personal history of a hereditary cancer syndrome - Suboptimal metagenomic read counts |
| Liu 2021^36^ | All Chinese | **Controls:**  Male – 50%  Female – 50%  **Adenoma:**  Male – 66.7%  Female – 33.3%  **CRC:**  Male – 50%  Female – 50% | **Controls:**  Mean – 37.0    **Adenoma:**  Mean – 59.1    **CRC:**  Mean – 60.8 | Not reported | Not reported | Not reported | Not reported | None reported |
| Lv 2023^40^ | All Chinese | **Controls:**  Male – 36.6%  Female – 63.4%    **Cases:**  Male – 54.8%  Female – 45.1% | **Controls:**  Mean – 52.46  SD – 8.261  **Cases:**  Mean – 50.57  SD – 6.841 | **Controls:**  Mean – 22.53  SD – 3.00  **Cases:**  Mean – 23.45  SD – 2.93 | **Controls:**  Smokers – 11.3%  Non-smokers – 88.7%  **Cases:**  Smokers – 9.3%  Non-smokers – 90.7% | **Cases:**  Hypertension – 9.7%  **Controls:**  Hypertension – 11.3% | Not reported | - History of gastrointestinal surgery - Functional or metabolic bowel lesions within the past 3 months - Had taken medication for infectious diseases in the past 1 month - Had undergone GI endoscopy within the past 6 months - Had taken probiotics in the past 1 month - A history of familial adenomatous polyposis and inflammatory bowel disease |
| Nakatsu 2018^26^ | All from Hong Kong, no further detail reported | **Controls:**  Male – 53.2%  Female – 46.7%  **Cases:**  Male – 64.8%  Female – 35.1% | **Controls:**  Mean – 65.51  SD – 7.79  **Cases:**  Mean – 72.50  SD – 11.04 | **Controls:**  Mean – 23.77  SD – 3.70  **Cases:**  Mean – 23.99  SD – 3.21 | **Controls:**  Chronic smokers – 0.0%  Non-smokers – 2.2%    **Cases:**  Chronic smokers – 2.7%  Non-smokers – 27.0% | **Controls:**  T2DM – 16.30%    **Cases:**  T2DM – 39.19% | Distal – 17.57%  Proximal – 82.43% | As per Yu 2017 |
| Tarallo 2019^44^ | Not reported | **Controls:**  Male – 54.1%  Female – 45.8%    **Adenoma:**  Male – 59.3%  Female – 40.7%    **CRC:**  Female – 20.7%  Male – 79.3% | **Controls:**  Mean –67.9  SD – 7.1  **Adenoma:**  Mean – 62.8  SD – 8.6  **CRC:**  Mean – 71.4  SD – 8.2 | **Controls:**  Mean – 25.3  SD – 3.5    **Adenoma:**  Mean – 25.3  SD – 4.1    **CRC:**  Mean – 25.7  SD – 4.1 | Not reported | Not reported | Not reported | - Hereditary CRC syndromes - Previous history of CRC - Uncompleted or poorly cleaned colonoscopy - Use of antibiotics during the 6 months before sample collection |
| Touchefeu 2020^45^ | Not reported | **Controls:**  Male – 15%  Female – 85%    **Cases:**  Male – 61.9%  Female – 38.1% | **Controls:**  Median – 58  Range – 46-79    **CRC:**  Median – 68  Range – 45-79 | **Controls:**  Median – 25.5  Range – 19.1-36.1    **CRC:**  Median – 25.2  Range – 18.5-33.6 | Not reported | Not reported | Right – 38.1%  Left – 33.3%  Rectum – 28.6% | - IBD - Irritable bowel syndrome - Immunosuppressive therapy or corticosteroids >10 mg - Antibiotic, prebiotic or probiotic therapy within the previous month - Bowel preparation within 1 week - Chemotherapy or radiotherapy for the treatment of CRC - Control subjects with previous history of cancer |
| Vogtmann 2016^49^ | **Cases:**  Non-Hispanic White – 75%  Non-Hispanic Black – 23.1%  Other – 1.9%    **Controls:**  Non-Hispanic White – 90.4%  Non-Hispanic Black – 5.8%  Other – 3.8% | **Controls:**  Male – 71.2%  Female – 28.8%    **Cases:**  Male – 71.2%  Female – 28.8% | **Controls:**  Mean – 61.2  SD – 11.0    **Cases:**  Mean –61.8  SD - 13.6 | **Controls:**  Mean – 25.3    **Cases:** Mean – 24.9 | **Controls:**  Never smoked – 42.3%  Former smokers – 53.8%  Current smokers – 8.3%  Missing – 0%    **Cases:**  Never smoked – 46.2%  Former smokers – 34.6%  Current smokers – 13.5%  Missing – 5.8% | Not reported | Right – 46.2%  Left – 34.6%  Rectal – 26.9%  Missing – 9.6% | - Adenomatous polyps or - non-neoplastic bowel - conditions following - surgery or pathology - review |
| Yachida 2019^11^ | Not reported | **Controls:**  Male – 55.9%  Female – 44.1%    **Polyps:**  Male – 71.7%  Female – 28.4%    **Stage 0:**  Male – 61.7%  Female – 38.4%    **Stage I/II:**  Male – 65.8%  Female – 34.2%    **Stage III/IV:**  Male – 58.1%  Female – 41.9% | **Controls:**  Mean – 64.65  SD – 10.55    **Polyps:**  Mean – 63.15  SD – 9.12    **Stage 0:**  Mean – 64.82  SD – 7.54    **Stage I/II:** Mean – 63.76  SD – 9.32    **Stage III/IV:**  Mean – 59.09  SD – 10.99 | **Controls:**  Mean – 22.98  SD – 2.77    **Polyps:**  Mean – 23.09  SD – 4.7    **Stage 0:**  Mean – 23.33  SD – 3.57    **Stage I/II:**  Mean – 22.83  SD – 3.17    **Stage III/IV:**  Mean – 23.06  SD – 3.16 | Not reported | Not reported | **Stage 0:**  Left colon – 31.9%  Right colon – 43.1%  Left and right colon –4.2%  Rectum – 20.1%    **Stage I/II:**  Left colon – 29.7%  Right colon – 28.8%  Left and right colon –1.8%  Rectum – 39.6%    **Stage III/IV:**  Left colon – 18.3%  Right colon – 28.2%  Left and right colon –2.8%  Rectum – 50.7%    (Location of polyps not reported) | - Insufficient stool sample - Hereditary or suspected hereditary disease (FAP, HNPCC, MSI High) - IBD - Abdominal surgical history |
| Yang 2020^32^ | Not reported | **Controls:**  Male – 47.3%  Female – 52.7%  **Cases:**  Male – 67.3%  Female – 32.7% | **Cases:** Mean – 53 Range – 33-74    **Controls:**  Mean – 42  Range – 20-72 | **Controls:**  Median – 23.21  Range – 19.87-27.6    **Cases:**  Median – 23.49  Range – 17.51-28.73 | Not reported | Not reported | Not reported | All participants:   - ≤18 years old or ≥76 years old - Colorectal benign lesion - Antibiotics exposure - Radiotherapy - Continuous treatment by systemic corticosteroids 1 month prior to sampling - Serious mental disorder   Healthy controls only:   - Dysentery - Chronic enteritis - Inflammatory bowel disease - Irritable bowel syndrome, - Metabolic diseases (BMI ≥ 32, diabetes or malnutrition) - Long-term probiotics uptake |
| Yang 2021^37^ | All from China – no other detail reported | **oControls:**  Male – 50%  Female – 50%  **oCRC:**  Male – 74.0%  Female – 26.0%  **yControls:**  Male – 46.0%  Female – 54.0%  **yCRC:**  Male – 54.0%  Female – 46.0% | **oControl:**  Mean – 63.36  SD – 9.67  **oCRC:**  Mean – 63.58  SD – 8.29  **yControl:**  Mean – 40.76  SD – 6.09  **yCRC:**  Mean – 40.66  SD – 6.69 | Not reported | Not reported | Not reported | **yCRC:**  Right – 24%  Left – 28%  Rectum – 48%  **oCRC:**  Right – 14%  Left – 30%  Rectum – 56% | CRC group:   - History of familial CRC - History of inflammation-associated CRC - History of irritable bowel syndrome (IBS) - Other co-existing malignant tumours - Stool sampling not done before colonoscopy - Neoadjuvant therapy before stool sampling   Control group:   - Gastrointestinal tumours after colonoscopy screening |
| Yu 2017^25^ | All from China – no other detail reported | **Controls:**  Male – 61.1%  Female – 38.9%    **Cases:**  Male – 64.9%  Female – 35.1% | **Controls:**  Median – 63  Range – 50-73    **Cases:**  Median – 67  Range – 34-89 | **Controls:**  Median – 22.9  Range – 17.1-35.1    **Cases:**  Median – 23.9  Range – 17.3-31.3 | Not reported | **Controls:**  T2DM **–** 29.6%    Total cholesterol – median 5.0, range 3.2-6.7    eGFR – median 69.51, range 50.82-115.04    **Cases:**  T2DM – 39.2%    Total cholesterol – median 4.9, range 2.6-8.6  eGFR – median 71.13, range 16.81-136.52 | Not reported | - Use of antibiotics in last 3 months - Vegetarian diet - Invasive medical intervention within 3 months - History of any cancer - Inflammatory bowel disease |
| Zeller 2014^42^ | Not reported | Male – 58.4%  Female – 41.5%    (data for cases and controls not given separately) | **Controls:**  Mean – 60.5    **Small adenoma:**  Mean – 60.3    **Large adenoma**:  Mean – 67.7    **CRC:**  Mean – 66.8 | **Controls:**  Mean – 25    **Small adenoma:**  Mean – 25    **Large adenoma:**  Mean – 26    **CRC:**  Mean – 27 | Not reported | Not reported | **CRC:** Rectum – 17.0% Sigmoid – 7.5% Left colon – 43.4%  Right colon – 32.1%  **Large adenoma:** Rectum – 6.7% Left colon – 40% Right colon – 26.7% Left and right – 26.7%  **Small adenoma:**  Rectum – 22.2% Sigmoid – 18.5% Left colon – 22.2% Right colon – 29.6% Left and right – 7.4% | - Previous colon or rectal surgery - Colorectal cancer - Inflammatory or infectious injuries of the intestine - Patients with need for emergency colonoscopy |
| Zhang 2023^41^ | All from China – no other detail reported | Male – 64.3%  Female – 35.7%    (data for cases and controls not given separately) | **Controls:**  Mean – 54.3  **Cases:**  Mean – 56.3 | **Controls:**  Mean – 20.3  **Cases:**  Mean – 22.7 | **Controls:**  Never smoked – 50%  Ex smokers – 16.7%  Current smokers – 33.3%  **Cases:**  Never smoked – 50%  Ex smokers – 25%  Current smokers – 25% | **Controls:**  Hypertension – 0%    Diabetes – 0%    **Cases:**  Hypertension – 0%    Diabetes – 0% | Ascending – 63%  Transverse – 25%  Descending – 13% | - History of previous CRC or IBD - Radiotherapy or chemotherapy - Gastrointestinal surgery within three months - Antibiotics or probiotic preparations within three months - Colonoscopy within one month |
| Zhang 2022^39^ | Not reported | **Controls:**  Male – 57.1%  Female – 42.9%    **Adenoma:**  Male – 58.6%  Female – 41.4%    **CRC:**  Male – 66.7%  Female – 33.3% | **Controls:**  Mean – 57.53  SD – 7.70  **Adenoma:**  Mean – 56.71  SD – 8.21  **CRC:**  Mean – 57.82  SD – 9.30 | **Controls:**  Mean – 23.09  SD – 2.39  **Adenoma:**  Mean – 24.01  SD – 2.60  **CRC:**  Mean – 22.51  SD – 2.38 | **Controls:**  Current smokers – 28.57%  Not current smokers – 71.43%  **Adenoma:**  Current smokers – 24.14%  Not current smokers – 75.86%    **CRC:**  Current smokers – 26.67%  Not current smokers –73.33% | **Controls:**  Hypertension – 22.86%    Diabetes – 11.43%    **Adenoma:**  Hypertension – 34.48%    Diabetes – 13.79%    **CRC:**  Hypertension –16.67%    Diabetes – 16.67% | **CRC:**  Colon 53.3% Rectum 46.7%    (Location of polyps not reported) | - Age under 18 - Pregnancy - Infection - Chronic inflammation - Mental disease - Gastrointestinal surgery - Previous history of other colonic diseases - Use of antibiotics within the past 2 months - Refusal to participate after reading the informed consent form |

*Supplementar*y *Table 6: Additional methodology (including handling of stool samples, laboratory methods and bioinformatic analysis)*

| **Study** | **Stool sampling method** | **Sampling time relative to colonoscopy and bowel preparation** | **Sample storage method** | **Sample storage time** | **DNA extraction technique** | **Library preparation technique** | **Sequencing platform** | **Read depth** | **Annotation database used** |
| --- | --- | --- | --- | --- | --- | --- | --- | --- | --- |
| Avelar-Barragan 2022^47^ | Zymo DNA/RNA shield preservation buffer (R1101) | 4-6 weeks post colonoscopy | Posted by patients at room temperature, then frozen at -80°C on arrival in laboratory | Not reported | Using Zymo kit according to manufacturer’s instructions | Illumina DNA prep kit (20018705) using the author’s published low volume protocol | Illumina HiSeq 4000 | Average of 1,102,247 ± 643,325 high-quality, non-human reads per sample | - R v3.6.3 - Vegan v2.5-6 |
| Bucher-Johannessen 2023^46^ | Fresh frozen stool | Before sigmoidoscopy, participants collected samples at home and stored for up to 7 days in a domestic freezer (−20°C) prior to delivery to the screening centre | Stored at screening centre at −20°C | 17 years (archived samples) | QIAsymphony automated extraction system using a QIAsymphony DSP Virus/ Pathogen Midi Kit | Riptide protocol (Twist Bioscience HQ, CA, USA) | Illumina NovaSeq | Median read depth of 6.2 million reads (0.76-20.1) | - MetaPhlAn 3 - HUMAnN 3 |
| Chang 2021^33^ | Fresh stool | Not reported | Transported to laboratory within 2 hours and frozen at -40°C | Not reported | CWBIO Stool Genomic DNA Kit (CW2092, CWBIO, China) | Not reported | Illumina HiSeq 2500 | Not reported | - Kraken 2.0 - Braken 2.5 - HUMAnN 2 |
| Coker 2019^27^ | Not reported | Not reported | Stored at −20°C (domestic freezer) within 4 hours post defaecation and then at −80°C (laboratory freezer) within 24 hours | Not reported | Qiagen QIAmp DNA Stool Mini Kit (Qiagen) | Not reported | Illumina HiSeq 2000 | Average of 27,640 420 high-quality paired reads per sample (5Gb) | - Kraken - A custom database (from NCBI, FungiDB, Ensemble and Broad Institute) was built using Jellyfish |
| Coker 2020^28^ | As per Coker 2019 | As per Coker 2019 | As per Coker 2019 | As per Coker 2019 | As per Coker 2019 | As per Coker 2019 | As per Coker 2019 | As per Coker 2019 | As per Coker 2019 |
| Coker 2022^38^ | Not reported | Not reported | Stored at −20°C (domestic freezer) within 4 hours post defaecation and then at −80°C (laboratory freezer) within 24 hours | Not reported | Qiagen QIAmp DNA Stool Mini Kit (Qiagen) | Not reported | Illumina HiSeq 2000 | Average of 2,316,872 ± 267,563 (rarefied to 1,947,705 - the minimum read count of all samples) | - MetaPhlAn 2 |
| Feng 2015^43^ | Fresh stool samples, mechanically homogenized with a sterile spatula and split into aliquots | Not reported | Faecal aliquots were stored at home in domestic freezers (−20 °C) and transported to the laboratory within 48hrs in a freezer pack, where they were immediately stored at −80 °C | Not reported | Not reported | Not reported | Illumina | Not reported | - IMG database and an in house pipeline |
| Gao 2020^31^ | Fresh stool on ice | Not reported | Delivered within 2 hours to the laboratory and then each sample was divided into 3-5 aliquots in sterile tubes and stored in a -80°C freezer | Not reported | E.Z.N.A.® DNA Kit (Omega Bio-tek, Norcross, USA) | Not reported | Illumina HiSeq 4000 | Not reported | - BLASTP - NCBI NR database - eggNOG |
| Gao 2021^34^ | Fresh stool on ice | Mixed pre and post colonoscopy samples  (Ratio before:after - Cancer 43:28, Adenoma 28:35, Controls 40:51) | Frozen at -80°C immediately on arrival in laboratory (within 2 hours of voiding) | Not reported | QIAamp Fast DNA Stool Mini Kit (Qiagen, Hilden, Germany) | Illumina TruSeq DNA Sample Prep v2 Guide | Illumina HiSeq | An average of 9.49 ± 3.74Gb of ‘clean’ data per sample | - Kraken - BLASTP - KEGG |
| Gao 2022^35^ | As per Gao 2021 | As per Gao 2021 | As per Gao 2021 | As per Gao 2021 | As per Gao 2021 | As per Gao 2021 | As per Gao 2021 | As per Gao 2021 | As per Gao 2021 |
| Gupta 2019^29^ | Stool collected in 'sterile containers' | Prior to colonoscopy (bowel preparation not reported) | Immediately after collection samples were transported to the laboratory at 4°C where they were frozen at -80°C | DNA was extracted immediately after receiving samples, then frozen at -80 until sequencing (not reported how long frozen as DNA) | QIAamp stool minikit (Qiagen, CA, USA) | Illumina Nextera XT sample preparation kit | Illumina NextSeq 500 | Mean of 1.35Gb per sample | - Human Microbiome Project-National Center for Biotechnology Information (HMP-NCBI) - De novo clustering-based metagenomic species (MGS) - Clade-specific-marker-based metagenomic OTU (mOTU) species - Metaphlan - KEGG - eggNOG |
| Hannigan 2018^48^ | Fresh stool in a container with no preservatives | Participants provided their stool sample between 1-2 weeks after their colonoscopy and bowel preparation | Frozen at -80°C (no other details) | Not reported | Genomic DNA was extracted from purified virus-like particles (VLPs) from stool samples, using a modified version of a previously published protocol | Illumina Nextera XT preparation kit | Illumina HiSeq 4000 | Not reported | - ‘A reference database consisting of all bacteriophage and eukaryotic virus genomes present in the European Nucleotide Archives’ was used |
| Lee 2023^50^ | Stool samples were collected in 100% ethanol and aliquoted | 'Stool samples were not collected at the time of colonoscopy to avoid potential effects of the bowel preparation' – but exact timeframe not reported | Stored at -80°C (no other details) | Not reported | Allprep DNA/RNA Mini Kit (Qiagen) | Nextera XT DNA Library Preparation Kit (Illumina) | Illumina HiSeq 2500 | Approx. 2.5Gb per aliquot | - MetaPhlan 3.0 - HUMAnN 3 |
| Liu 2021^36^ | Sterile 5-ml tubes containing 2ml DNA/RNA shield liquids (DeepBiome Co., Ltd) | Not reported | Not reported | Not reported | DNeasy PowerSoil Kit (Qiagen, Germany) | KAPA HyperPrep Kit (Illumina; KK8504) | Illumina | Approx. 1 Gb per sample | - Kraken 2 |
| Lv 2023^40^ | Fresh stool | Not reported | Samples were kept at 4°C after defecation and transported to the laboratory within 12h of defecation (in a container filled with liquid nitrogen) where they were frozen at -80°C | Not reported | Detailed protocol described in text | Library preparation was completed by terminal repair, adding A tail, adding sequencing joint, purification, PCR amplification and other steps | Illumina Novaseq | Not reported | - Kraken2 - Braken - Self-built microbial nucleic acid database (NCBI NT nucleic acid database and RefSeq whole genome database) |
| Nakatsu 2018^26^ | Not stated | Not stated | Stored at −20°C (domestic freezer) within 4 hours post defaecation and then at −80°C (laboratory freezer) within 24 hours | Not reported | Qiagen QIAamp DNA Stool Mini Kit according to the manufacturer’s instructions | Illumina (as per manufacturer’s instructions) | Illumina HiSeq 2000 | Not reported | - Kraken |
| Tarallo 2019^44^ | Stool nucleic acid collection and transport tubes with RNA stabilizing solution (Norgen Biotek Corp) | Prior to bowel preparation and colonoscopy (specific timeframe not reported) | Samples handed in at time of colonoscopy (not reported how stored prior), then frozen at -80C until extraction | Not reported | QIAamp DNA stool minikit (Qiagen, Germany) | Nextera XT DNA library preparation kit | Illumina HiSeq 2500 | Average 5gb per sample | - MetaPhlan 2.0 - Human 2 |
| Touchefeu 2020^43^ | Not reported | Participants were excluded if they had bowel preparation within one week prior to sampling (no other details reported) | Samples were collected at hospital and stored immediately at −80°C | DNA extracted in March 2017 (no other details reported) | AllPrep DNA/ RNA Mini Kit (Tissue DNA Isolation Kit; MO BIO Laboratories, Carlsbad, California, USA) | Not reported | Illumina HiSeq 2000 | Average 4.9 Gb (ranging between 7.8 and 24.1 million reads) | - MetaPhlAn 2.0 - Human 2 |
| Vogtmann 2016^49^ | Stool collected in a ‘plastic container’ | ‘Prior to surgery or other treatment, participants collected all stools over a two day period’ (no other details reported) | After collection stored on dry ice at patient’s home, then freeze-dried in laboratory and stored at -40°C | Freeze dried for >30 years | GNOME® DNA Isolation Kit (MP Biomedicals) with minor modifications | Library preparation was automated and adapted on a Biomek FXp Dual Hybrid (Beckman Coulter, Brea, USA), and a robotic PCR cycler (Biometra, Göttingen, Germany) | Illumina HiSeq 2000/2500 | Average 5Gb | - MOCAT |
| Yachida 2019^11^ | Not reported | Collected at first bowel movement after bowel preparation initiated | After collection stored on dry ice, then frozen at -80°C after DNA extraction | Not reported | GNOME® DNA Isolation Kit (MP Biomedicals). | Nextera XT DNA sample Prep Kit (Illumina, San Diego, USA) | Illumina HiSeq 2000/2500 | Average 49,375,231 paired end reads in total, 5.0 Gb (44,227,127 high quality reads) | - SILVA database - Validated using mOTU profiler and MetaPhlAn2 |
| Yang 2020^32^ | Not reported | Stool for CRC patients were collected ‘in the early morning after admission to the hospital’ (not reported if this is after bowel preparation). Stools from healthy patients were collected at time of physical examination in the hospital | All collected samples were frozen on dry ice within 30min of defaecation and then stored at −80°C until further analysis | Not reported | QIAamp DNA Stool Mini Kit (QIAGEN, 19593) | Illumina | Illumina HiSeq | Average of 37.56 million reads per sample | - Integrated Gene Catalog (IGC database) using Bowtie2 |
| Yang 2021^37^ | Stool collected in a ‘sterile tube’ | Before colonoscopy (no other details reported) | Frozen at -80°C (no other details reported) | Not reported | QIAamp DNA Stool Mini Kit (Qiagen, Germany) | TruSeq Nano DNA LT Library Preparation Kit (Illumina) | Illumina NovaSeq6000 | Not reported | - NCBI NR database by DIAMOND (v0.9.14) - Kraken2 (v2.1.1) - Braken (v2.5) - GO - KEGG |
| Yu 2017^25^ | Stool collected in a ‘standardised container’ | Both pre and post colonoscopy  Controls: Median -1.5 days (Range -34 days to +106 days)  Cases:  Median 19.9 days (-111 days to +2  days) | Collected at home and frozen in home freezer, delivered to hospital in insulated containers then frozen at -80°C once received | Controls: Median -185.1 days (range 86.7 to 2032 days)  Cases:  Median 149 days (range 6.7 days to 1280 days | Qiagen QIAamp DNA Stool Mini Kit | Illumina | Illumina HiSeq 2000 | Average high quality, non-human reads - Cases: 58112890 ± 10324458 Controls: 59380535 ± 7378751 | - Integrated Microbial Genome (IMG) reference database (v400) - In house pipeline |
| Zeller 2014^42^ | Fresh stool | Samples collected 2 weeks to 3 days prior to colonoscopy (prior to bowel preparation) | Stool was frozen at −20°C within 4hrs of defaecation and deposited at the hospital biobank | Not reported | GNOME® DNA Isolation Kit (MP Biomedicals) with minor modification | Biomek FXp Dual Hybrid | Illumina HiSeq 2000/2500 | 5Gb | - MOCAT - Vegan R package - Also used an internal pipeline as validation |
| Zhang 2023^41^ | Fresh stool collected in sterile collection tubes | Not reported | Collected in the hospital and snap-frozen in liquid nitrogen, then stored at -80°C | DNA extracted within one month | QIAamp DNA stool Mini Kit (Qiagen) | Not reported | Not reported | Not reported | - RDP and Silva databases - Mothur - BLASTP - DIAMOND KEGG - CAZy - Uniprot |
| Zhang 2022^39^ | Fresh stool | Prior to colonoscopy (no other details) | Frozen in a −80 ◦C refrigerator within 4hrs of defaecation | Not reported | TIANamp Stool DNA Kit protocol | DNA Sequencing Kit (NEXTflex, US) | Illumina NovaSeq 6000 | 42,574,669 ± 2,214,780 raw reads per sample | - MetaPhlAn3 - Kraken2 (viruses) |

*Supplementary Table 7: Complete version of Table 2 (containing cancer stages for Yachida et al. and F.nucleatum subspecies where reported)*

| **Bacterial species** |  | **Chang 2021**^33^ | **Coker 2022**^38^ | **Feng 2015**^43^ | **Gao 2020**^31^ | **Gao 2021**^34^ | **Gupta 2019**^29^ | **Tarallo 2019**^44^ | **Vogtmann 2016**^49^ | **Yachida 2019**^11^ | **Yang 2020**^32^ | **Yang 2021**^37^ | **Yu 2017**^25^ | **Zeller 2014**^42^ | **Zhang 2022**^39^ |
| --- | --- | --- | --- | --- | --- | --- | --- | --- | --- | --- | --- | --- | --- | --- | --- |
| *Fusobacterium nucleatum* | *Subspecies unspecified* | p=0.0078653713 | BH adj p<0.05 |  | Bonferroni adj p<0.05 | BH adj p<0.05 |  |  | p=0.04291649792 |  | BH adj p=0.000558 |  | BH adj p=0.000156 |  | BH adj p<0.05 |
|  | *Subspecies animalis* |  |  |  |  |  |  |  |  | BH adj p=0.0001545 (Stage I/II) |  |  |  | FDR adj p=0.0000751 |  |
|  |  |  |  |  |  |  |  |  |  | BH adj p=0.00000597 (Stage III/IV) |  |  |  |  |  |
|  | *Subspecies fusiforme* |  |  |  |  |  |  |  |  | BH adj p=0.00296803 (Stage I/II) |  |  |  |  |  |
|  |  |  |  |  |  |  |  |  |  | BH adj p=0.02890365 (Stage III/IV) |  |  |  |  |  |
|  | *Subspecies nucleatum* |  |  |  |  |  |  |  |  | BH adj p=0.04919493 (Stage 0) |  |  |  | FDR adj p=0.000654 |  |
|  |  |  |  |  |  |  |  |  |  | BH adj p=0.0000563 (Stage I/II) |  |  |  |  |  |
|  |  |  |  |  |  |  |  |  |  | BH adj p=0.000000262 (Stage III/IV) |  |  |  |  |  |
|  | *Subspecies polymorphum* |  |  |  |  |  |  |  |  | BH adj p=0.04919493 (Stage 0) |  |  |  | FDR adj p=0.0032 |  |
|  |  |  |  |  |  |  |  |  |  | BH adj p=0.00011515 (Stage I/II) |  |  |  |  |  |
|  |  |  |  |  |  |  |  |  |  | BH adj p=0.000048 (Stage III/IV) |  |  |  |  |  |
|  | *Subspecies vincentii* |  |  |  |  |  |  |  |  | BH adj p=0.0000906 (Stage I/II) |  |  |  | FDR adj p=0.00003 |  |
|  |  |  |  |  |  |  |  |  |  | BH adj p=0.00000211 (Stage III/IV) |  |  |  |  |  |
| *Parvimonas micra* |  | p=0.0011513806 | BH adj p<0.05 | Bonferroni adj p=0.0000 | Bonferroni adj p<0.05 | BH adj p<0.05 | FDR adj p=0.000110447834 |  |  | BH adj p=0.0103511 (Stage I/II) |  |  | BH adj p=0.0000000543 |  | BH adj p<0.05 |
|  |  |  |  |  |  |  |  |  |  | BH adj p=0.00000000466 (Stage III/IV) |  |  |  |  |  |
| *Gemella morbillorum* |  | p=0.004802717073 | BH adj p<0.05 | Bonferroni adj p=0.0000 | Bonferroni adj p<0.05 |  |  |  |  | BH adj p=0.00061526 (SI/II) | BH adj p=0.000086 |  |  |  | BH adj p<0.05 |
|  |  |  |  |  |  |  |  |  |  | BH adj p=0.00000211 (Stage III/IV) |  |  |  |  |  |
| *Peptostreptococcus stomatis* |  |  | BH adj p<0.05 | Bonferroni adj p=0.0000 | Bonferroni adj p<0.05 |  | FDR adj p=0.0000159 |  |  | BH adj p=0.00321513 (Stage I/II) |  |  |  | FDR adj p=0.022 | BH adj p<0.05 |
|  |  |  |  |  |  |  |  |  |  | BH adj p=0.000000375 (Stage III/IV) |  |  |  |  |  |
| *Prevotella intermedia* |  |  | BH adj p<0.05 |  | Bonferroni adj p<0.05 | BH adj p<0.05 |  |  |  | BH adj p=0.04799026 (Stage III/IV) |  |  |  |  | BH adj p<0.05 |
| *Solobacterium moorei* |  |  | BH adj p<0.05 |  | Bonferroni adj p<0.05 |  |  | BH adj p=3.60E-02 |  | BH adj p=0.03813521 (Stage 0) |  |  | BH adj =0.00985 |  |  |
|  |  |  |  |  |  |  |  |  |  | BH adj p=0.01948575 (Stage III/IV) |  |  |  |  |  |
| *Bacteroides fragilis* |  |  | BH adj p<0.05 |  |  | BH adj p<0.05 | FDR adj p=0.0000187 |  |  |  |  |  |  |  | BH adj p<0.05 |
| *Clostridium symbiosum* |  |  | BH adj p<0.05 | Bonferroni adj p=0.0002 |  |  |  | BH adj p=4.49E-02 |  |  |  |  |  |  | BH adj p<0.05 |
| *Escherichia coli* |  |  |  | Bonferroni adj p=0.0013 |  | BH adj p<0.05 | FDR adj p=0.0004981816723 | BH adj p=7.65E-03 |  |  |  |  |  |  |  |
| *Filifactor alocis* |  |  | BH adj p<0.05 |  | Bonferroni adj p<0.05 |  |  |  |  | BH adj p=0.00400351 (Stage I/II) | BH adj p=0.00006 |  |  |  |  |
|  |  |  |  |  |  |  |  |  |  | BH adj p=0.01983532 (Stage III/IV) |  |  |  |  |  |
| *Fusobacterium varium* |  |  | BH adj p<0.05 |  |  | BH adj p<0.05 |  |  |  |  | BH adj p=0.048714 |  |  |  | BH adj p<0.05 |
| *Parabacteroides distasonis* |  |  |  | Bonferroni adj p=0.0016 |  |  | FDR adj p=0.00002005 |  |  |  |  | p<0.05 (old onset) |  |  | BH adj p<0.05 |
| *Porphyromonas asaccharolytica* |  |  | BH adj p<0.05 |  |  | BH adj p<0.05 |  |  |  |  | BH adj p=0.021559 |  |  | FDR adj p=0.00961 |  |
| *Clostridium bolteae* |  |  | BH adj p<0.05 | Bonferroni adj p=0.0000 |  |  |  |  |  |  |  |  |  |  | BH adj p<0.05 |
| *Clostridium ramosum* |  |  | BH adj p<0.05 |  | Bonferroni adj p<0.05 |  |  |  |  |  | BH adj p=0.000674 |  |  |  |  |
| *Flavonifracter plautii* |  |  |  |  |  |  | FDR adj p=0.000000000672 |  |  |  |  | p<0.05 (young onset) |  |  | BH adj p<0.05 |
| *Fusobacterium mortiferum* |  |  |  |  |  |  |  | BH adj p=3.61E-02 |  | BH adj p=0.02827984 (Stage I/II) |  |  |  |  | BH adj p<0.05 |
| *Porphyromonas gingivalis* |  |  |  |  | Bonferroni adj p<0.05 | BH adj p<0.05 |  |  |  |  | BH adj p=0.021559 |  |  |  |  |
| *Prevotella nigrescens* |  |  |  |  | Bonferroni adj p<0.05 |  |  |  |  |  | BH adj p=0.010417 |  |  | FDR adj p=0.0215 |  |

Appendix 3: Glossary of terms

| **Term** | **Definition** |
| --- | --- |
| 16s rRNA amplicon sequencing | A form of next generation, or massively parallel, sequencing which amplifies the 16s rRNA marker gene (present in all bacteria and archaea) which can then be mapped to annotation databases to identify the organisms present. Performed on automated sequencing machines. Sometimes referred to as ‘16s sequencing’ or ‘amplicon sequencing’. |
| Alpha diversity | Alpha diversity refers to the diversity of microbial species within a single sample. It measures both the number of different species present (richness) and how evenly those species are distributed (evenness) within that sample. Measures include Shannon, Simpson and Chao indices. |
| Annotation database/pipeline | Computational tools which convert raw DNA sequences into taxonomic or functional data, providing information on the genes and taxa present in a sample. Examples include MetaPhlAn and Kraken. |
| Beta diversity | Beta diversity measures the differences in species composition between different samples (in contrast to alpha diversity which measures diversity within a single sample). Measures include Bray-Curtis dissimilarity, Jaccard index, and UniFrac distance. |
| DNA extraction | A laboratory process by which all the DNA contained in a sample (such as faeces) is isolated and purified. This may contain human, bacterial, viral and other types of DNA. The pure DNA can then be processed further, e.g. sequencing. |
| Functional profile | The breakdown of which genes are present in a sample (can only be achieved with shotgun rather than 16s sequencing). Provides an insight into how the organisms may behave, e.g. genes for toxin formation (although does not provide information on whether these genes are being actively expressed). |
| Library preparation | The process of converting DNA or RNA into a ‘library’ of fragments that can be sequenced, usually done on automated machines. This involves fragmenting the DNA/RNA, adding sequencing adapters, and potentially amplifying the library. The goal is to create a pool of fragments of a suitable size and with adapter sequences that are compatible with the specific sequencing platform being used. |
| Read depth | The number of times a specific base (nucleotide) in a DNA sequence is read during the sequencing process. Higher read depths generally lead to more accurate and reliable sequencing results. |
| Taxonomic profile | The breakdown of which organisms are present in a sample, usually presented to genus or species level, however strain level is also possible with shotgun sequencing. |
| Whole metagenome shotgun sequencing | A form of next generation, or massively parallel, sequencing which involves untargeted or ‘shotgun’ sequencing of all DNA present in a sample. The sequences identified can then be mapped to annotation databases. Performed on automated sequencing machines. Sometimes referred to as ‘shotgun sequencing’, ‘shotgun metagenomics’ or ‘metagenomics’. |
